# A Layered Framework for Biological Aesthetics: A Narrative Review of Regenerative Medicine in Cosmetic Science

**DOI:** 10.7759/cureus.115688

**Published:** 2026-09-02

**Authors:** Mohamed Ismail, Scott M Martin

**Affiliations:** 1 Regenerative Aesthetics, Clinica Joelle Dubai, Dubai, ARE; 2 Medical Aesthetics, Private Practice, Las Vegas, USA

**Keywords:** aesthetics, exosomes, mesenchymal stem cells, multi-layered treatment, platelet-rich fibrin, regenerative medicine

## Abstract

Biological aesthetics applies regenerative substances, including platelet-rich fibrin, mesenchymal stem cells, and fat-derived biological matrices, with emerging adjunctive roles for peptides and exosomes. These approaches have gained attention because of their potential regenerative and biocompatible properties. However, comparative safety, clinical effectiveness, and durability remain uncertain because the available evidence is heterogeneous and, for several modalities, limited to preclinical studies or small clinical investigations. A layered approach addressing bone, fascia, fat, dermis, and epidermis is proposed as a framework for studying age-related changes across multiple tissue planes, but it has not been validated as a clinical treatment protocol. Challenges remain regarding regulatory clarity, product standardization, dosing, safety, and long-term clinical validation. This narrative review summarizes the biological basis, available clinical evidence, regulatory limitations, and research gaps associated with major regenerative agents and presents the multilayered model as a hypothesis-generating framework for future research.

## Introduction and background

The growing demand for youthful and naturally rejuvenated skin has accelerated the evolution of minimally invasive cosmetic techniques focused on regeneration and repair [1]. While synthetic agents such as botulinum toxin and hyaluronic acid fillers offer rapid cosmetic improvement, their temporary effects and known complication profiles have contributed to increasing interest in biologically integrated alternatives, although comparative safety and long-term efficacy remain under investigation [1,2].

This shift toward biological aesthetics reflects a growing need for interventions that engage native tissue physiology to support repair, regeneration, and long-term structural improvement rather than temporary correction.

Biological aesthetics focuses on reversing the signs of skin aging through advanced biological toolkits, including platelet-rich plasma (PRP), platelet-rich fibrin (PRF), stem cells, exosomes, autologous fat, peptides, growth factors, and specialized devices. These regenerative agents enhance natural tissue repair and regeneration, providing a biologically compatible approach that may complement or, in selected cases, offer an alternative to synthetic interventions. Their integration into modern aesthetics reflects the growing influence of regenerative medicine in cosmetic science [3-5]. Biological aesthetics focuses on restoring soft tissues that have been lost or damaged by the aging process. By revitalizing the skin's intrinsic architecture and stimulating collagen synthesis, biological aesthetics aims to support longer-term structural and functional outcomes rather than solely superficial correction [6]. These biologically derived substances can range widely, from sophisticated molecules made by biotechnology to those found in cells. For example, adipose tissue contains mesenchymal stem cells (MSCs) that can differentiate into various cell types, including fat and skin cells, and can be utilized in aesthetic medicine [2,7-10]. Further emerging perspectives in biological aesthetics involve multilayered treatments that target multiple tissue layers, including bone, fat, fascia, dermis, and epidermis, to address age-related changes in the face and achieve more thorough, natural-looking rejuvenation [11,12]. This method acknowledges that each layer ages uniquely and that treating them separately could yield inconsistent outcomes. Practitioners can achieve a more youthful, beautiful appearance by considering the interrelationships among these layers [13]. This layered framework follows a clinical logic: restore the foundation first (bone → fascia → fat → dermis → epidermis), ensuring that deeper structures are stabilized before refining surface features.

Despite growing interest in regenerative aesthetic interventions, the available literature remains fragmented. Individual studies generally evaluate specific agents, frequently using preclinical models, small patient samples, heterogeneous preparations, short follow-up periods, and nonstandardized outcomes (Figure 1). There is limited comparative evidence showing whether multilayer combinations provide greater efficacy, safety, or durability than established aesthetic treatments. Furthermore, no prospectively validated protocol currently defines how biological interventions should be selected or sequenced across different facial tissue layers. This gap limits the translation of biological plausibility into evidence-based clinical recommendations.

**Figure 1:**
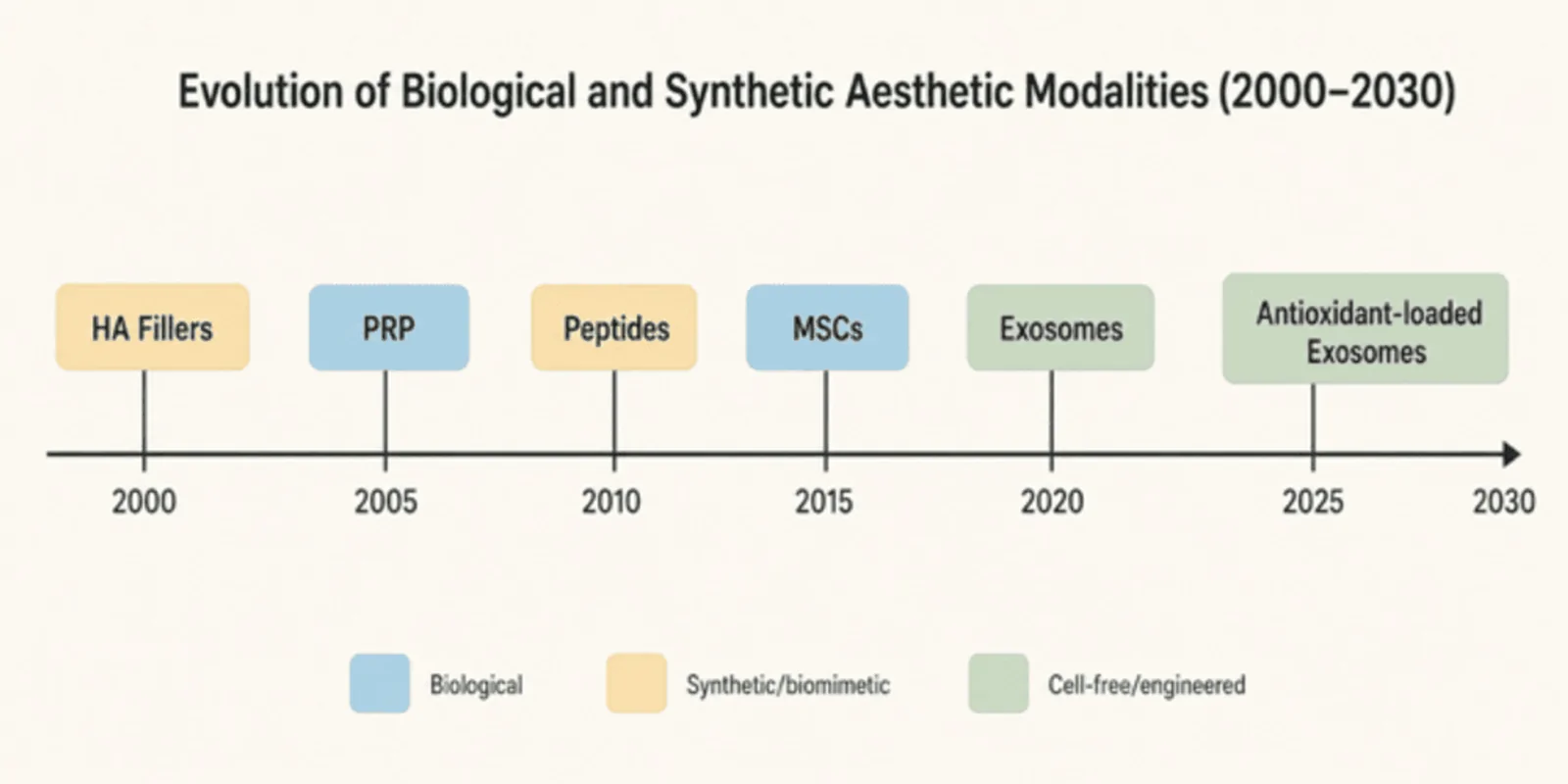
Evolution of Biological vs. Synthetic Aesthetic Modalities (2000-2030) This timeline conceptually illustrates the chronological evolution of aesthetic interventions from synthetic agents to biologically regenerative methods. The progression reflects growing clinical interest in biocompatibility, longevity of results, and cellular-level rejuvenation. Data are compiled from major reviews in aesthetic dermatology and from market analyses of the regenerative medicine market [1–5]. HA, hyaluronic acid; MSCs, mesenchymal stem cells; PRP, platelet-rich plasma. This author-developed conceptual timeline was created using BioRender (trial version, Science Suite Inc. d/b.a. BioRender, Toronto, Canada) for graphical elements and Microsoft PowerPoint (Microsoft Corporation, Redmond, Washington) for arrangement, labeling, and formatting. Images courtesy of Dr. Mohamed Ismail.

Methods of literature identification and narrative synthesis

This article was designed as a narrative review and conceptual synthesis rather than a systematic review or meta-analysis. Targeted searches of PubMed/MEDLINE, Google Scholar, ClinicalTrials.gov, and relevant regulatory websites were conducted through the time of writing. Search terms included combinations of “regenerative aesthetics,” “platelet-rich fibrin,” “platelet-rich plasma,” “mesenchymal stem cells,” “exosomes,” “extracellular vesicles,” “bioactive peptides,” “fat grafting,” “nanofat,” “facial aging,” and “skin rejuvenation.” Reference lists of relevant reviews and primary studies were also screened.

Evidence was interpreted according to study design. Systematic reviews and controlled human studies were given greater weight than observational studies and case series, while in vitro and animal studies were used only to describe biological plausibility. Clinical trial registration and manufacturer information were not considered evidence of clinical efficacy unless supported by published clinical results. Because the review used a purposive narrative approach rather than an exhaustive systematic search, no meta-analysis or formal risk-of-bias assessment was performed, and a PRISMA (Preferred Reporting Items for Systematic Reviews and Meta-Analyses) workflow was not applied. 

## Review

Proposed layered biological aesthetic framework

Age-related anatomical changes occur across bone, fat, fascia, muscle, dermis, and epidermis. The following layered framework organizes candidate regenerative modalities according to their proposed anatomical targets and biological mechanisms. It is based primarily on anatomical literature, preclinical findings, early clinical studies, and expert interpretation. It has not been prospectively validated, and no comparative study has established that treating deeper layers first, combining multiple biological agents, or following a particular sequence improves efficacy, safety, or durability. The framework should therefore be interpreted as a structure for research and discussion rather than a clinical treatment protocol.

The layered biological aesthetic protocol emphasizes restoration from within, targeting each facial tissue layer to improve both function and appearance (Figure 2). By modifying and enhancing the biological processes of the skin, this method is designed to improve the sustainability and natural appearance of outcomes, although long-term comparative data remain limited [12-20]. Like rebuilding a house from its foundation upward, true rejuvenation begins by restoring deeper structures before refining surface details [14]. Figure 3 illustrates the imbalance between biological treatment priorities and global spending on aesthetics, highlighting an overinvestment in surface procedures relative to deeper regenerative interventions. Currently, people tend to spend more on lower-priority features, such as lip enhancements and surface treatments, while paying less attention to high-impact foundational structures, such as bone and muscle. However, targeting deeper biological layers is hypothesized to contribute to more natural and potentially longer-lasting results, although this relationship has not been fully quantified. Although precise quantitative data are lacking, clinical experience and anatomical studies suggest that more profound structural changes (e.g., bone resorption, deflation of the fat compartments, ligamentous loosening) drive a substantial portion of visible facial aging. 

**Figure 2:**
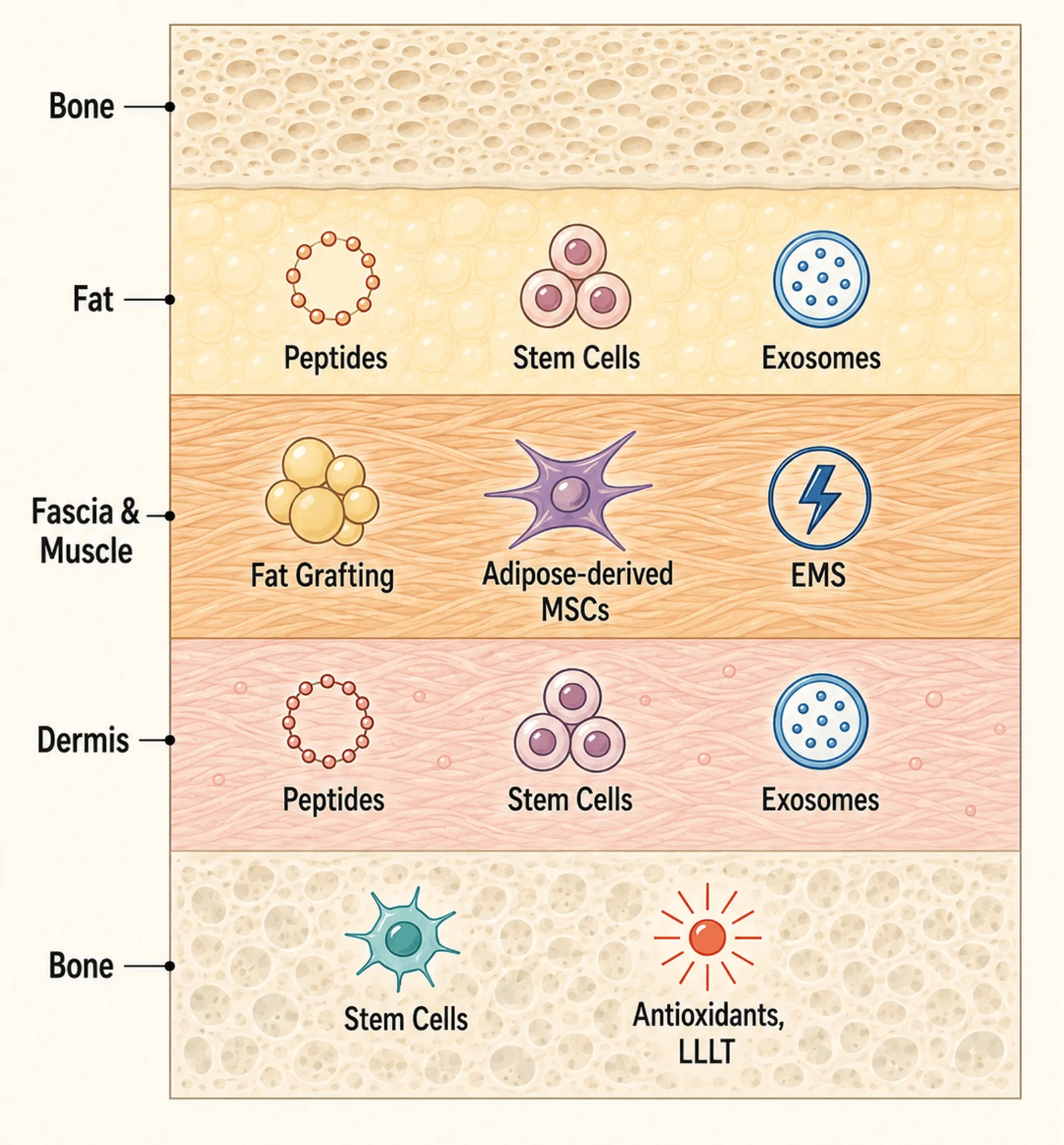
Author-Proposed Layered Research Framework for Biological Aesthetic Modalities This schematic representation illustrates the multi-layered approach in biological aesthetics, integrating regenerative techniques tailored to each anatomical stratum. MSC-based therapies (including Wharton’s jelly-derived MSCs) target bone support; fat matrix and nanofat restore volumetric and regenerative function; PRF enhances dermal angiogenesis; while epidermal therapies include adjunctive exosomes and antioxidants. Adapted from recent clinical and anatomical frameworks [12–19]. This author-developed schematic organizes candidate regenerative modalities according to the anatomical tissue layers they are proposed to affect. The associations are based on anatomical, mechanistic, preclinical, and early clinical literature. The figure does not represent a validated treatment sequence, clinical decision tree, dosing protocol, or recommendation to combine the illustrated interventions. The placement of a modality within a tissue layer does not indicate established efficacy, superiority, or regulatory approval for aesthetic use. MSCs: mesenchymal stem cells; PRF: platelet-rich fibrin. This figure was created using BioRender (trial version, Science Suite Inc. d/b.a. BioRender, Toronto, Canada) for graphical elements and Microsoft PowerPoint (Microsoft Corporation, Redmond, Washington) for arrangement, labeling, and formatting. Images courtesy of Dr. Mohamed Ismail.

**Figure 3:**
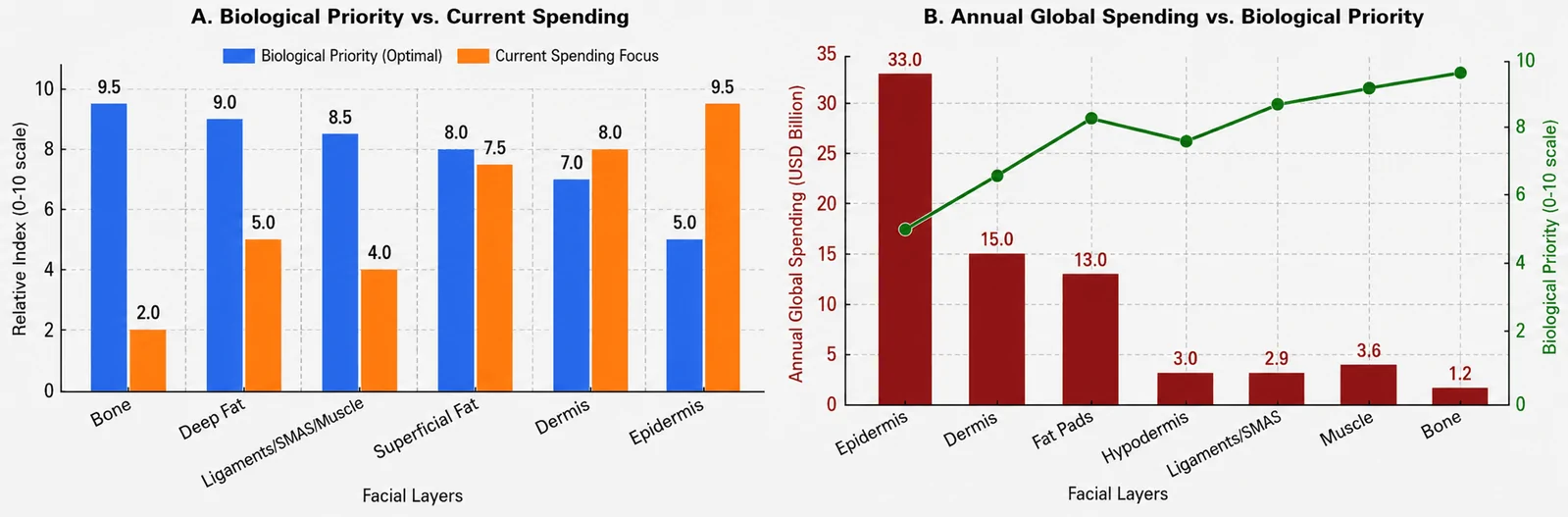
Layered Biological Aesthetics: Comparative Perspectives on Aging and Investment (A) Relationship between biological priority and current treatment spending across facial layers, highlighting the under-investment in deeper regenerative structures. (B) Annual global aesthetic spending (maroon bars, USD billion) contrasted with biological relevance (green line, 0–10 scale) across facial layers. Both panels are conceptual, synthesized from anatomical frameworks discussed by de Sanctis Pecora et al. [20] and supported by clinical market-trend data in regenerative aesthetics. The figure was prepared using Microsoft PowerPoint 2010 (Microsoft Corporation, Redmond, Washington).

In contrast, a significant portion of aesthetic spending is directed toward superficial layers, such as the dermis and epidermis. This misalignment may contribute to less durable outcomes [14,21]. Future studies should evaluate whether matching an intervention to a specific anatomical tissue layer improves clinically relevant outcomes. Layer-specific interventions have biological plausibility, but their comparative clinical efficacy and durability have not been established. This anatomical organization provides a framework for examining candidate interventions across individual tissue planes.

Each biological component, including MSCs, exosomes, PRF, peptides, and fat matrix, acts synergistically through paracrine communication, growth factor signaling, and extracellular matrix remodeling.

Bone layer

The facial skeleton provides structural support and defines contour; age-related bone resorption contributes to sagging and loss of definition. Sagging or a loss of definition can result from age-related or other changes in bone structure. When planning treatments such as dermal filler insertion or bone grafting to restore volume and support, practitioners must have a thorough understanding of bone function and its potential for resorption [14,15]. Sagging and loss of contour are examples of changes in facial features that occur as bone density declines with aging, since bone structure serves as the foundation of the face. Although individuals with weak facial skeletal support are more likely to experience early signs of aging, those with strong skeletal features offer good support to the soft tissue that lies on top of them and are linked to late clinical symptoms of age-related bone loss [22]. Bone remodeling is a lifelong process that is dynamically balanced across life stages and in response to demands [23]. Several bioactive peptides have demonstrated osteogenic effects in laboratory and preclinical studies. However, their role in facial skeletal rejuvenation remains experimental, and clinical evidence supporting aesthetic application is currently unavailable.

Peptides that bind to BMP type I receptors, such as the D5 peptide, can also promote osteoblast development, mineralization, and proliferation. These approaches are being investigated for their potential role in bone remodeling and may have indirect effects on facial structural support [24]. Stem cells promote tissue regeneration and represent a potential strategy under investigation to address bone-related volume loss, particularly in cosmetic medicine [25,26].

Emerging sources such as Wharton’s jelly-derived MSCs offer enhanced proliferative capacity and immunomodulatory properties, making them promising candidates for deep structural regeneration in aesthetic medicine.

Fat layer

The face's fat layer is essential for preserving its volume, youthful features, and curves. It is divided into superficial and deeper pockets, with the deep layer located below the superficial musculoaponeurotic system and the superficial layer positioned immediately beneath the skin. For treatments such as fat grafting, fillers, and the treatment of age-related volume loss, it is therefore crucial to understand these layers [15]. Autologous fat is considered a valuable regenerative option due to its biocompatibility and potential for long-term integration for facial rejuvenation because it is 100% biocompatible, produces a soft, natural result, and can be a permanent intervention [16]. As a result, dermal filler injections and fat grafting have become increasingly popular as complementary treatments for facial rejuvenation procedures in recent years [17,27]. It is crucial to determine the optimal depth and position of the fat pockets to receive the grafts. The superficial musculoaponeurotic system divides the superficial and deep fat spaces, the two main fat compartments of the face. Therefore, to maximize the benefits of fat grafting and minimize the risk of complications, a basic understanding of facial anatomy and the interactions between different structures and fascial planes is essential [25,26]. The nasolabial fat pad, the temporal and forehead fat pads, the superior, inferior, and lateral orbital fat pads, the jowl fat pad, and the medial, middle, and lateral cheek fat pads are typically found in the superficial fat area. The buccal fat pad, the deep medial and lateral cheek fat pads, the sub-orbicularis oris fat pad, and the medial and lateral suborbicularis oculi fat pads make up the deep fat space [19]. By understanding these units, injectors can select optimal sites for fat transfer [16]. Adipose stem cells, which are present in fat tissue, have a significant impact on aesthetic medicine and are increasingly used in cosmetic surgeries, such as breast augmentation, scar removal, and facial rejuvenation. Their capacity to differentiate into diverse cell types, such as blood vessels and skin cells, facilitates tissue repair and improves the appearance of the treated area [28]. Adipose-derived stem cells within grafted fat enhance survival through angiogenic and paracrine effects, improving texture and elasticity. MOTS-c has primarily been evaluated in experimental research on metabolism and aging. Its clinical effectiveness for modifying facial adipose tissue or producing aesthetic improvement has not been established [29].

Comparison of fat grafting, nanofat, stromal vascular fraction, and synthetic fillers

Conventional fat grafting, nanofat, stromal vascular fraction (SVF), and synthetic fillers are not interchangeable treatments. Conventional fat grafting transfers autologous adipose tissue containing mature adipocytes and stromal cells and is used primarily for volume restoration. It may provide lasting correction in surviving grafted tissue, but retention is variable, and the procedure requires harvesting, processing, and reinjection of fat.

Nanofat is produced through mechanical processing, emulsification, and filtration of harvested adipose tissue. This processing disrupts many mature adipocytes; therefore, nanofat is generally investigated for potential effects on skin quality, scars, fine rhytides, and pigmentation rather than dependable volumetric augmentation. A systematic review identified possible improvements in scars and skin rejuvenation but found that the included studies were heterogeneous and generally provided low-level evidence.

SVF is a heterogeneous cellular component of adipose tissue that includes stromal, vascular, immune, and progenitor cells. It may be administered with fat grafts to investigate whether cellular enrichment improves graft retention. Although some studies report improved volume persistence, the effects of isolation methods, cellular composition, dosing, regulatory classification, and long-term outcomes remain inconsistent. Consequently, SVF-assisted fat grafting remains an emerging approach rather than an established aesthetic standard.

Synthetic fillers, including hyaluronic acid, calcium hydroxylapatite, and poly-L-lactic acid products, have standardized commercial formulations and do not require a donor-site procedure. They generally provide more predictable initial correction, although their duration, reversibility, biological effects, and adverse event profiles differ by material. Hyaluronic acid fillers can be treated with hyaluronidase, whereas autologous fat cannot be removed as predictably. Both synthetic fillers and fat injections carry procedure-related risks, including vascular occlusion, contour irregularity, nodules, infection, and inflammatory reactions.

A randomized comparison of hyaluronic acid and autologous fat for nasolabial fold correction found similar outcomes during the first nine months, with differences emerging at 12 months. However, direct comparative evidence remains limited, and the available studies do not establish the overall superiority of one approach. Treatment selection therefore requires indication-specific assessment rather than assuming that biological materials are inherently safer, more durable, or more effective than synthetic fillers.

Fascia and muscle

The fascial system, a network of connective tissue, and its interaction with the muscles in the face and body are the main topics of the facial layer aesthetic procedure. By understanding how these layers interact and influence appearance, this method aims to address aesthetic concerns. The superficial and deep fascial layers, as well as the superficial musculoaponeurotic system that connects the skin to the facial muscles, are recognized in the layered biological aesthetic protocol. It acknowledges how muscles and fascia interact to sustain general tissue, create volume, and produce facial expressions. Tightness or imbalances in these structures can cause wrinkles, sagging, and other cosmetic problems [30]. Due to its inherent regenerative qualities, PRF is used in cosmetic procedures, such as muscle-layer treatments [31,32]. Growth factors such as PDGF, TGF-beta, and VEGF are found in PRF within muscles and fascia. Essentially, these factors stimulate fibroblast activity, leading to increased synthesis of collagen and elastin. This can enhance the overall appearance of the muscular layer and the skin's flexibility and hardness. Moreover, PRF's growth factors promote cell migration, differentiation, and proliferation, which aid in tissue regeneration and repair within the muscle layer and reduce the visibility of fine lines and wrinkles, especially where there is underlying muscle tone [33]. Exosomes are being investigated in muscle-layer protocols for their potential to increase collagen and elastin production and have been associated with improvements in skin elasticity and appearance in early studies [34]. Muscle hypertrophy (increased size and strength) and better muscle definition can result from these contractions [35]. These technologies may enhance muscle tone, improve soft tissue positioning, and support structural longevity when combined with regenerative therapies.

Dermis

The dermis layer is the primary target of many aesthetic medicine therapies designed to heal scars, reduce wrinkles, and rejuvenate the skin. Targeting the dermis and subcutaneous layers, injectable fillers add volume beneath the skin's surface to improve facial features, reduce wrinkles, and restore volume. This method involves making microscopic skin punctures to stimulate collagen and elastin production. The dermis can be targeted with a variety of lasers, which increase collagen production and treat issues such as wrinkles, UV damage, and pigmentation. Deeper peels can reach the dermis to treat more severe wrinkles, scars, and pigmentation problems, even though some peels target only the epidermis [36]. Exosomes are becoming increasingly important in aesthetic medicine and cosmetics. Exosomes affect collagen synthesis and reduce inflammation and pigmentation. Additionally, they reverse fibroblast senescence and promote the formation of extracellular matrix components, thereby facilitating overall skin regeneration [37]. A concentrated mixture of platelets, growth factors such as PDGF, VEGF, and TGF-β, and other advantageous cells found in PRF, which is made from the patient's own blood, stimulates collagen synthesis and acts as a sustained-release biological scaffold, promoting angiogenesis, fibroblast activation, and extracellular matrix remodeling, thereby supporting dermal regeneration, contributing to improvements in skin texture, and promoting general skin rejuvenation [38-40]. Preclinical studies and small product-specific clinical investigations suggest that GHK-Cu may influence collagen synthesis and extracellular matrix remodeling. However, evidence regarding its independent clinical effect on wrinkles, skin density, and pigmentation remains limited [41].

Epidermis

In aesthetic medicine and cosmetics, red lasers are primarily used for skin rejuvenation and to treat superficial issues such as wrinkles, fine lines, and pigmentation problems, as they directly target the epidermis, the outermost layer of the skin. They give the appearance of being tighter, smoother, and younger by promoting the development of collagen and elastin [42,43]. A series of biological processes is initiated when red light lasers, especially low-level laser therapy (LLLT), penetrate the epidermis and are absorbed by cellular components, such as mitochondria, thereby increasing collagen and elastin production, which are essential for skin firmness and suppleness [42,44]. Exosomes and oligopeptides are emerging adjunctive therapies in epidermal rejuvenation; however, their clinical application remains limited by variability in preparation and dosing and by a lack of long-term standardized evidence. The ability of topical exosomes to support epidermal regeneration is showing promise in cosmetic medicine by providing bioactive chemicals that promote cell regeneration, reduce inflammation, and enhance skin suppleness and texture [37]. They can regulate pigment synthesis and influence melanocyte migration, with potential effects on pigmentation and skin brightness [37,45]. Free radicals are unstable molecules that can damage skin cells and contribute to the premature aging of the skin. Many skincare products contain antioxidants, such as vitamin C and vitamin E, as well as plant-derived extracts, which help fight free radicals and prevent premature aging, supporting a healthy complexion [46]. Certain antioxidants, such as vitamin C, can help lighten the skin and reduce the appearance of hyperpigmentation by preventing melanin production [47,48]. Niacinamide in the epidermis is crucial for aesthetic procedures, as it enhances skin health and addresses various skin concerns. It aids in protein synthesis, hydration retention, and skin protection against environmental harm [49]. It promotes ceramide synthesis in the skin, which is essential for preserving hydration and enhancing its barrier function [49,50].

The biological toolkit: key regenerative agents

While peptides and exosomes have gained significant attention, their clinical translation remains under investigation, with limitations in standardization and long-term outcome data.

The biological toolkit in regenerative aesthetics comprises a spectrum of biomaterials and bioactive agents designed to restore or replace aged or damaged tissue, harnessing the body’s intrinsic repair mechanisms [51]. The basic idea behind biological toolkits used in aesthetics is to rejuvenate and regain a youthful appearance by utilizing the body's inherent healing and regenerative capabilities. It deviates from conventional aesthetic techniques, as this discipline offers a more natural and potentially longer-lasting method of tissue-level rejuvenation and aesthetic enhancement by utilizing biological processes to replace or repair tissues damaged by aging or other factors [52-57]. Among various regenerative materials, stem cell-derived factors, platelet concentrates, and bioactive peptides are currently the focus of significant clinical interest. The following subsections summarize their biological basis, aesthetic relevance, and evidence. Figure 4 illustrates the primary regenerative mechanisms of major biological agents used in aesthetic medicine.

**Figure 4:**
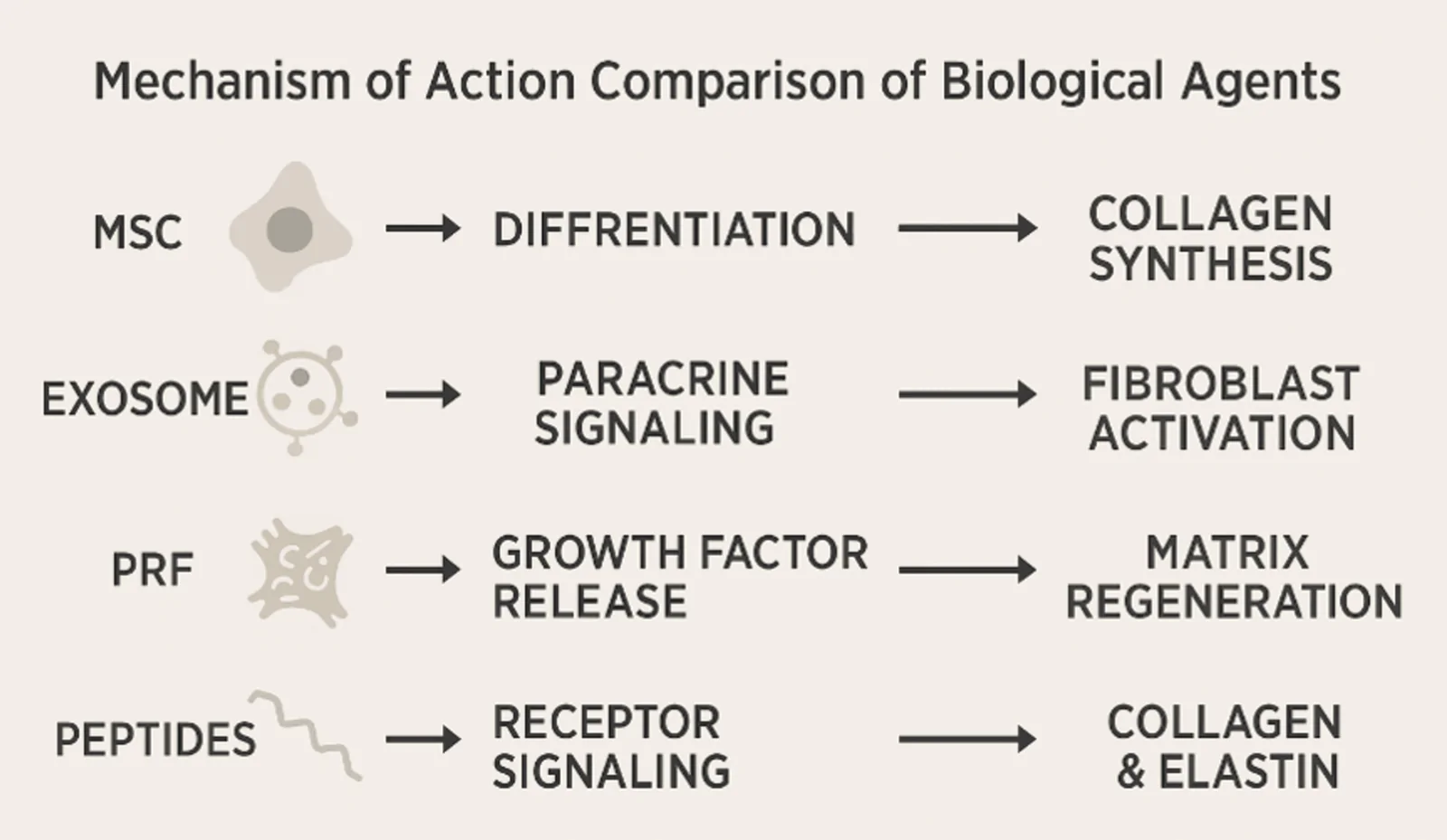
Mechanism of Action Comparison of Key Biological Aesthetic Agents This figure illustrates the primary regenerative mechanisms of major biological agents used in aesthetic medicine. MSCs facilitate tissue regeneration through differentiation and collagen synthesis; exosomes act via paracrine signaling and fibroblast activation; PRF releases growth factors that promote matrix remodeling; and bioactive peptides trigger receptor-mediated collagen and elastin synthesis. Adapted and synthesized from current molecular and clinical evidence [51–57]. The pathways shown illustrate biological plausibility and should not be interpreted as evidence of comparative clinical efficacy. MSCs: mesenchymal stem cells; PRF: platelet-rich fibrin. This figure was created using BioRender (trial version, Science Suite Inc. d/b.a. BioRender, Toronto, Canada) for graphical elements and Microsoft PowerPoint (Microsoft Corporation, Redmond, Washington) for arrangement, labeling, and formatting. Images courtesy of Dr. Mohamed Ismail.

Mesenchymal stem cells

MSCs, first identified in bone marrow and later isolated from adipose tissue and the umbilical cord, are multipotent progenitors with demonstrated regenerative potential in preclinical and early clinical studies [58]. In bioaesthetics, MSCs are showing promise, especially for wound healing and skin regeneration. These multipotent cells, which can differentiate into various cell types, including those crucial to skin health such as fibroblasts and endothelial cells, are found in multiple organs, including bone marrow and adipose tissue. MSCs are being investigated for their potential to improve skin healing in injured or aging skin, lower inflammation, and stimulate the formation of collagen and elastin in aesthetic applications [9]. They have shown promising effects in experimental and early clinical settings across several skin-related in vitro and in vivo studies, helping alleviate skin conditions and improve skin appearance [10].

Adipose tissue-derived stem cells

Adipose tissue-derived stem cells (ADSCs) are the most commonly used because they are readily obtained and exhibit strong therapeutic efficacy. Adipose tissue is separated from the lipoaspirate to extract ADSCs. Unlike ADSCs, bone marrow stem cells are difficult to obtain, and the process is frequently unsuccessful [53,59,60]. Due to their ease of extraction, ADSCs have gained significant popularity recently, and researchers in aesthetic medicine have devoted the most attention to them [56,57]. Adipose stem cells and stromal vascular fraction (SVF), which is made up of fibroblasts, macrophages, smooth muscle cells, and endothelial cells, make up adipose tissue. ADSCs are separated from the SVF via enzymatic digestion. Mixing with fetal bovine serum, filtration, and centrifugation are the subsequent stages of extraction [61,62]. Under specific conditions, ADSCs can develop into endodermal or ectodermal cells. ADSCs are more resistant to apoptosis and have a higher survival rate than BM-SCs. Multipotent activity, paracrine effects, and immunomodulatory effects are the foundations of their mode of action [63]. The administration of SVF is the most straightforward approach to application. However, due to necrosis and cell aggregation, the application is ineffective. Researchers are currently focusing on developing culture systems with ADSCs to enhance efficacy. There are still numerous extraction procedures in use, and no standardized method exists for extracting and preparing fat grafts [53,59,63].

Umbilical cord-derived stem cells

HUC-MSCs are multipotent and self-renewing. Under certain circumstances, they can continually divide and differentiate into one or more of the cell types that comprise human tissues and organs. They are easily harvested, separated, cultivated, enlarged, and purified, and they affect immunological responses. HUC-MSCs maintain their stemness even after several expansions and passages. HUC-MSCs can be used in allografts since their surface antigens are not readily noticeable, transplanted cells are not often rejected, and matching requirements are not stringent [64].

HUC-MSCs are now being used to treat several aging-related issues, as they possess several unique qualities: (i) Development: The production of differentiated cells by HUC-MSCs enhances tissue function and promotes tissue regeneration [61]. (ii) Immune regulation: By secreting interleukin-10 (IL-10) and interleukin-4 (IL-4), HUC-MSCs suppress the growth of immune cells, including T cells, B cells, and Tfh cells. They also induce macrophages to shift from a proinflammatory to an anti-inflammatory phenotype, thereby reducing inflammation. These immune response alterations work together to promote tissue repair [65]. (iii) Paracrine effects: By releasing soluble molecules such as keratinocyte growth factor (KGF), hepatocyte growth factor (HGF), epidermal growth factor (EGF), and other cytokines, HUC-MSCs encourage tissue regeneration [65,66]. (iv) Anti-inflammatory effect: HUC-MSCs inhibit the release of inflammatory factors, including interleukin 1β (IL-1β), tumor necrosis factor-α (TNF-α), and interleukin-8 (IL-8), which reduces inflammation and oxidative stress and consequently inhibits cell apoptosis [67]. (v) Antifibrotic activity: HUC-MSCs promote the release of HGF and other cytokines, as well as cell death associated with fibrosis. The enhancement of vascular remodeling and the control of associated signaling pathways can also mediate the antifibrotic effect [68]. (vi) Regulation of noncoding RNA: HUC-MSCs can influence the expression of circular RNA (circRNA), long noncoding RNA (lncRNA), and microRNA (miRNA), which indirectly regulate their target genes and have therapeutic effects [69].

One prospective source of MSCs is the human umbilical cord (UC). In addition to their well-known benefits, which include a painless collection process and quicker self-renewal, UC-MSCs have demonstrated the capacity to differentiate into three germ layers, to proliferate in inflammatory or damaged tissue, to facilitate tissue repair, and to regulate the immune system [64]. Despite promising outcomes, variability in extraction and culture methods limits their standardization. Long-term safety data and regulatory clarity remain key barriers to the widespread adoption of aesthetic procedures (Table 1).

**Table 1:** Key Differences Between Adipose Tissue-Derived Stem Cells and Umbilical Cord-Derived Stem Cells UC-MSCs: umbilical cord-derived mesenchymal stem cells; ADSCs: adipose-derived stem cells.

Differences	Adipose tissue-derived stem cells	Umbilical cord-derived stem cells	References
Source and availability	Collected from adipose tissue (fat) through liposuction or other surgical procedures	Collected from the umbilical cord and Wharton's jelly after childbirth	[50]
Harvest ease	Can be obtained in relatively large quantities due to the abundance of adipose tissue	Limited by the volume of umbilical cord blood and tissue	[35]
Immunogenicity	These are more immunogenic	UC-MSCs are generally considered less immunogenic	[70]
Proliferation	Less proliferative	UC-MSCs tend to have a higher proliferative capacity (grow and divide more rapidly) than ADSCs	[71]
Invasiveness	Somewhat invasive	Non-invasive	[72]
Apoptosis tolerance	High	Not high	[72]

Exosomes

In the realm of aesthetics, exosomes, which are tiny, membrane-bound vesicles released by cells, play a key role in paracrine signaling, particularly in skin regeneration and renewal [70-73]. They serve as messengers, delivering a variety of bioactive substances, including proteins, lipids, and nucleic acids, that can affect recipient cell behavior and support antiaging effects, collagen synthesis, and wound healing [55]. Through various signaling channels, exosomes influence the behavior of recipient cells by delivering their contents. Numerous processes, including endocytosis, direct membrane fusion, and receptor-ligand interactions, enable cells to absorb exosomes. The biological effects of exosomes on recipient cells are determined by the particular molecules they carry, including cytokines, growth factors, and miRNAs [74,75]. By promoting angiogenesis (the development of new blood vessels), stimulating fibroblast migration and proliferation, and reducing inflammation, exosomes derived from various cells, including stem cells, may accelerate wound healing. By increasing collagen production, decreasing wrinkles, enhancing skin suppleness, and potentially reversing fibroblast senescence, the process of cellular aging, exosomes may influence pathways associated with skin aging [76]. By influencing the wound-healing process and promoting tissue regeneration, exosomes have demonstrated potential in modulating scar formation in early studies and improving scar appearance [67]. By influencing melanin synthesis and altering melanocyte activity (cells that produce pigment), exosomes can alter skin pigmentation [77]. However, clinical translation remains limited by a lack of standardized isolation, storage, and dosing protocols, as well as cost-effective manufacturing challenges.

Platelet-rich fibrin

An autologous biomaterial made from the patient's own blood, PRF promotes wound healing and tissue regeneration. Platelets and leukocytes, which are abundant in growth factors and cytokines, are concentrated in a fibrin matrix. In aesthetic medicine, these elements are essential for promoting tissue regeneration and healing [78]. Since PRF is made from the patient's own blood, there is no chance of an allergic reaction or the spread of illness. Tissue regeneration is facilitated by the fibrin matrix, which serves as a scaffold for cell migration and proliferation. Numerous growth factors, including VEGF, PDGF, bFGF, and EGF, are released by platelets, promoting tissue healing, blood vessel development, and cell proliferation [79].

Additionally, PRF contains white blood cells (leukocytes) that aid the healing process by performing immune-related functions [80]. PRF is widely used in tissue engineering and aesthetic practice, as it utilizes a fibrin matrix to deliver growth factors gradually and continuously. Unlike other platelet concentrates, such as PRP, this matrix, which is made from the patient's own blood, promotes tissue regeneration and healing over a longer time span by serving as a scaffold for cell growth and a reservoir for growth factors. PRF can be used to increase collagen production, smooth out wrinkles, and improve skin texture, giving the appearance of younger skin [81,82]. However, both PRP and PRF are derived from a patient's own blood and contain concentrated platelets; their composition and preparation differ, which affects their effectiveness (Table 2). Whereas PRP is produced with anticoagulants and releases growth factors more quickly, PRF is produced without anticoagulants, allowing a natural fibrin matrix to release growth factors gradually [83]. Despite promising biological mechanisms, standardized protocols and large-scale clinical validation remain necessary before widespread adoption.

**Table 2:** Characteristic Features of Platelet-Rich Fibrin and Platelet-Rich Plasma

Characteristics	Platelet-rich fibrin (PRF)	Platelet-rich plasma (PRP)	References
Preparation	PRF is prepared without anticoagulants, allowing for the formation of a natural fibrin matrix.	PRP is prepared with anticoagulants.	[83]
Growth factor release	PRF's fibrin matrix results in a slower, more sustained release of growth factors.	Releases growth factors more rapidly.	[83]
Composition	PRF includes white blood cells and stem cells within its fibrin matrix.	Both contain platelets and growth factors.	[84]
Wound healing	PRF's sustained release of growth factors is thought to be beneficial for wound healing and tissue regeneration, with a long-lasting effect.	Immediate release. Short-lived effect.	[85]
Potential benefits	It may offer advantages in certain clinical contexts for addressing deeper wrinkles, restoring volume, and promoting overall skin health due to its sustained release of growth factors.	It may be effective for promoting hair growth, rejuvenating the skin, and reducing hair loss.	[83]

Regeneration peptides

Regeneration peptides are used to support skin renewal and repair processes in cosmetic medicine. Examples include Argireline (acetyl hexapeptide-8), Matrixyl (palmitoyl pentapeptide), and GHK-Cu (copper peptide), which increase skin firmness, decrease wrinkles, and encourage collagen formation. To enhance skin health and appearance, these peptides are used in a variety of skincare products and treatments [56,84,85]. Due to its antiaging and skin-regenerating properties, the copper-binding peptide GHK-Cu is increasingly being utilized in cosmetics. It is well recognized for increasing the formation of collagen and elastin, enhancing the firmness and elasticity of the skin, reducing fine lines and wrinkles, and promoting general skin renewal. Additionally, it has anti-inflammatory and antioxidant properties [41].

A mitochondrial-derived peptide called MOTS-c is being investigated for its potential cosmetic benefits, particularly for skin health and antiaging. The generation of more collagen, the reduction of inflammation, and the possible prevention of age-related skin changes are being investigated for potential effects on skin appearance. To confirm these outcomes and develop safe, efficient methods for aesthetic applications, additional clinical trials are necessary as research continues [57]. The synthetic peptide Matrixyl, also known as palmitoyl pentapeptide-4, is used in cosmetic products for its antiaging and skin-rejuvenating properties. This "collagen-boosting" substance helps to improve the firmness, elasticity, and overall appearance of the skin by promoting the formation of collagen, elastin, and other vital proteins [86]. The topical, short-acting synthetic peptide acetyl hexapeptide-8, commonly known as Argireline, has gained popularity recently for its anti-wrinkle properties. This substance has become a more widely available substitute for botulinum neurotoxin. Argireline, sometimes referred to as "Botox in a bottle," reduces the appearance of wrinkles and expression lines by relaxing the facial muscles. It enhances the suppleness and firmness of the skin [87]. The synthetic peptide palmitoyl tetrapeptide-7, also known as palmitoyl tetrapeptide-3, is used in cosmetics, particularly in anti-inflammatory and antiaging treatments. It improves the firmness and suppleness of the skin, reduces inflammation, and minimizes the appearance of fine lines and wrinkles. It functions by promoting the synthesis of collagen and hyaluronic acid while suppressing cytokine production that drives inflammation. This peptide contributes to a more youthful appearance by lowering inflammation and skin damage [88]. Although these peptides are increasingly explored in cosmetic formulations, evidence for them is largely limited to preclinical and small-scale clinical studies.

Fat matrix

A processed form of adipose (fat) tissue, either autologous (derived from the patient's own body) or allografted (derived from a donor), is referred to as "fat matrix" in aesthetic medicine. It acts as a scaffold for the creation of new fat cells and tissue regeneration. In the treated area, this matrix, which often contains proteins, growth factors, and other extracellular matrix constituents, provides a structure that stimulates the body to generate its own fat cells, leading to tissue rejuvenation and volume restoration [89]. New fat cells can proliferate and integrate into neighboring tissue using the fat matrix as a scaffolding. To promote cell development and regeneration, it contains proteins, growth factors, and other naturally occurring substances found in adipose tissue. Fat matrix can be allografted (from a donor, as in products like Leneva or Renuva) or autologous (from the patient's own body, as in fat grafting) [90]. The desired body part, such as the hands, face, or other places losing volume, is injected with the fat matrix. Existing fat cells and stem cells can proliferate under the ideal conditions of the matrix. New fat cells are naturally produced by the body to replace the matrix over time, providing the treated area with volume and renewal. A natural and durable outcome is achieved when the newly produced fat melds with the existing tissue [89].

Because of its healing properties, nanofat, a processed form of autologous fat, is becoming increasingly popular in aesthetic medicine. It provides a natural alternative to conventional fillers and surgery for skin rejuvenation, scar therapy, and volume restoration. Harvested fat tissue is physically processed to produce nanofat, which is a concentrated supply of growth factors and stem cells. Microfat, produced during liposuction, is filtered and emulsified during this procedure. Nanofat is mainly utilized for its regenerative and tissue remodeling properties, as opposed to microfat, which is employed for volume restoration [91]. Nanofat is used to treat sun-damaged skin, reduce wrinkles, and improve skin texture. It may help reduce the visibility of scars, such as those from acne and burns. Nanofat can be used to correct mild volume loss and enhance the appearance of hollows or depressions, even if it does not produce as much volume as standard fat grafting.

Additionally, nanofat is being investigated for hand rejuvenation, hair regeneration, and the treatment of under-eye discoloration [92]. To improve facial features and restore lost volume, fat is extracted from one part of the body and injected into the face as part of a cosmetic treatment called fat grafting or facial volumization, utilizing a fat matrix. Using the patient's own tissue, this method minimizes the risk of adverse reactions and yields long-lasting, natural-looking results. This well-liked facial rejuvenation technique addresses concerns such as wrinkles, hollows, and a general loss of facial fullness [93]. To summarize, facial fat grafting is a versatile and effective method of facial rejuvenation that utilizes the patient's own fat to enhance facial features, soften wrinkles, and restore volume.

Clinical evidence of biological aesthetics

Available human evidence ranges from small uncontrolled studies to early-phase and registered clinical trials, with substantial variation in interventions, preparation methods, outcome measures, and follow-up duration.

Several research reports have shown that MSCs can enhance tissue regeneration, reduce inflammation, and increase collagen synthesis, thereby improving skin texture and appearance (Table 3). However, to increase their efficacy, MSCs are also being investigated in combination with nanomaterials and other treatments [94].

**Table 3:** Selected Registered Studies Involving Regenerative Agents Relevant to Skin, Wound Healing, and Aesthetic Research Registration indicates that a study was planned or initiated; it does not confirm completion, publication, clinical efficacy, or safety. Some listed studies involve non-aesthetic indications and are included only to illustrate the translational development of these agents. MSC: mesenchymal stem cell; AD-MSCs: adipose-derived mesenchymal stem cells; UC-MSCs: umbilical cord-derived mesenchymal stem cells; IV: intravenous; PASI: Psoriasis Area and Severity Index; TBSA: total body surface area; ALLO-ASC-DFU: allogeneic adipose-derived stem cell therapy for diabetic foot ulcers; CO₂: carbon dioxide; RF: radiofrequency; WJ-MSCs: Wharton's jelly-derived mesenchymal stem cells; PRP: platelet-rich plasma; PRF: platelet-rich fibrin; PEFA: pulsed electrical field activated.

Category	Study Type	Phase	Summary/objective	ClinicalTrials.gov ID
MSC	Interventional	I	Stem cells are used to treat heel-pad injuries by accelerating healing.	NCT04219657
Autologous Adipose-Derived MSCs	Interventional	I/II	Compared stromal vascular fraction vs adipose-derived MSCs in large scars or Cutis laxa.	NCT03887208
Allogeneic MSCs	Interventional	I/II	Evaluated the safety and efficacy of allogeneic MSC therapy for severe systemic sclerosis.	NCT02213705
Adipose-Derived MSCs	Interventional	I/II	Tested the safety and efficacy of AD-MSCs for moderate–severe psoriasis using the PASI score.	NCT03265613
Umbilical-Cord MSCs	Interventional	I	Studied UC-MSC secretome/explants for skin rejuvenation and aging.	NCT06217627
Allogeneic MSCs	Interventional	I	Safety of donor-derived MSC therapy for 2° burn wounds (<20% TBSA).	NCT02104713
Allogeneic MSCs	Interventional	I/II	Evaluated ALLO-ASC-DFU in dystrophic epidermolysis bullosa.	NCT02579369
Adult Human MSCs	Interventional	I	Tested IV autologous MSCs in chronic/moderate atopic dermatitis.	NCT02888704
Stem-Cell Secretome	Interventional	NA	Compared the fractional CO₂ laser vs microneedling delivery of AD-MSC secretome for facial rejuvenation.	NCT05508191
ALLO-ASC-DFU	Observational	IV	Follow-up safety of ALLO-ASC-DFU therapy over 23 months.	NCT03183726
Umbilical-Cord MSCs	Interventional	NA	Compared UC-MSCs vs sweat-gland cells for wound transplantation of extensive skin lesions.	NCT02669199
UC-MSCs (Wharton’s Jelly)	Interventional	I	Assessed topical WJ-MSC-derived exosomes in chronic diabetic foot ulcers.	NCT06812637
MSC-Derived Exosomes	Interventional	I	Tested the topical MSC-exosome ointment safety/tolerability in healthy adults.	NCT05523011
Exosomes	Interventional	NA	Evaluated BENEV Exosome Regenerative Complex+ after RF-microneedling for hair thinning.	NCT06571799
Exosomes	Interventional	III	Investigated foreskin-derived MSC exosomes for androgenetic alopecia and satisfaction outcomes.	NCT06539273
Exosomes	Interventional	NA	Studied plant-based exosome formulation for androgenetic alopecia.	NCT06930326
Exosomes + PRP	Interventional	II	Compared exosomes and PRP for scalp hair growth at monthly intervals.	NCT06239207
Platelet-Rich Fibrin (PRF)	Interventional	IV	Evaluated Vivostat-PRF efficacy in non-ischemic foot ulcer healing.	NCT00770939
Platelet-Rich Fibrin (PRF)	Interventional	I/II	Studied biochemical/histologic effects of PRF injection into skin.	NCT00956020
PRF Matrix	Interventional	III	Compared photos and hair-density data 1–6 months post-treatment.	NCT01590238
PRP vs Tranexamic Acid	Interventional	I	Compared PRP with intralesional tranexamic acid for melasma.	NCT05884151
PRP (PEFA-activated)	Interventional	I/II	Evaluated pulsed-electrical-field-activated PRP (PEFA-PRP) vs unactivated PRP for androgenetic alopecia.	NCT05348343
PRP + Keratinocytes	Interventional	I	Tested autologous keratinocytes in PRP for wound-donor-site healing.	NCT00856934
PRP for Hair Loss	Interventional	I/II	Evaluated the safety and efficacy of PRP application for androgenetic alopecia.	NCT03488108

PRF gel is a viable treatment for under-eye rejuvenation in cosmetic and aesthetic applications, according to clinical studies. Autologous PRF can increase collagen generation, enhance skin texture and volume, and potentially reduce the visibility of dark circles and wrinkles [95,96].

Exosomes are showing promise in clinical trials for cosmetic and aesthetic uses. Several studies suggest that they help with wound healing, hair regrowth, and skin renewal. Research has shown that acne scars heal more quickly and that skin metrics, including moisture, suppleness, and wrinkle reduction, improve [97]. Clinical outcomes of common biological toolkits in aesthetic practice are presented in Table 4. 

**Table 4:** Clinical Outcomes of Common Biological Toolkits in Aesthetic Practice The categories above describe the stage and design of the available evidence and are not formal GRADE certainty ratings. The current evidence does not support standardized doses, injection volumes, treatment intervals, or maintenance schedules for these biological interventions. GRADE: Grading of Recommendations Assessment, Development and Evaluation.

Modality	Highest evidence represented in this review	Current interpretation	Main evidence gaps
PRP/PRF	Small clinical trials, observational studies, and reviews [78–83,95,96]	Early clinical support exists for selected dermatologic and aesthetic indications	Variable preparation, platelet composition, dosing, outcomes, and limited long-term comparative data
Autologous fat and nanofat	Clinical series and narrative reviews [89–93]	Fat grafting is clinically used for volume restoration; regenerative claims remain less certain	Variable graft retention, processing methods, patient selection, and limited controlled comparisons
MSC-based interventions	Preclinical studies and early-phase or registered clinical studies [58–69,94]	Experimental for aesthetic indications	Manufacturing variability, cell characterization, long-term safety, and regulatory restrictions
Exosome or extracellular-vesicle products	Predominantly preclinical studies, small clinical studies, and registered trials [70–77,97]	Experimental; current evidence does not support standardized routine use	Product heterogeneity, purity, dosing, storage, independent efficacy data, and long-term safety
Bioactive peptides	In vitro research and small product-specific studies [41,56,57,84–88]	Evidence is formulation-specific and should not be generalized across all peptides	Skin penetration, formulation differences, small samples, and lack of comparative trials

Patient personalization with genetic/epigenetic testing

In aesthetic medicine, patient personalization increasingly utilizes genetic and epigenetic testing to tailor treatments for optimal outcomes. To forecast skin aging patterns, treatment responses, and potential issues, these tests examine each person's unique genetic and epigenetic profiles. This enables the creation of individualized treatment programs tailored to the specific needs of certain patients, thereby maximizing results in areas such as scar management, antiaging treatments, and skin rejuvenation [98]. Genetic testing examines a person's DNA to determine aging trends, skin condition predispositions, and treatment response. Epigenetic testing explores how genes are expressed, demonstrating how environmental and lifestyle factors influence skin health and the aging process. Knowing methylation risk, inflammatory scores, and biological age may help tailor therapy regimens for optimal outcomes [20].

Skin health and healing may be impacted by the body's inflammatory condition, as indicated by inflammation scores. Methylation risk scores, particularly those based on DNA methylation patterns, can indicate a predisposition to disease and age-related changes. When considered collectively, these elements enable more focused and successful cosmetic treatments [99]. Customizing exosomes and peptides for individualized cosmetic treatments involves modifying these bioactive compounds to target specific skin issues and personal needs. To enhance the efficacy of exosomes and peptides for skin rejuvenation, antiaging, and the treatment of pigmentation disorders, this strategy focuses on improving their distribution and composition [20,73,99]. By examining how genes are expressed or not, without altering the DNA sequence itself, epigenetic mapping offers a novel approach to predicting aesthetic traits. Researchers may predict and potentially influence aesthetic outcomes by mapping these epigenetic markers and identifying patterns associated with specific aesthetic qualities [100]. Mapping these changes across the genome is known as epigenetic mapping. Patterns of gene expression associated with characteristics or circumstances are visible on this map. These alterations are identified and mapped using tools such as chromatin immunoprecipitation sequencing (ChIP-seq) and DNA methylation arrays [101]. Researchers can locate specific epigenetic signature patterns associated with aesthetic traits by examining epigenetic maps. For instance, specific methylation patterns may be linked to facial characteristics, hair texture, or skin pigmentation. Based on a person's epigenetic profile, predictive models can be created to assess the likelihood of specific aesthetic outcomes [74,101].

Safety, efficacy, and standardization

MSCs [102], exosomes [55], PRF, and fat-derived materials are generally well tolerated in controlled settings when handled under GMP or minimally manipulated conditions. Autologous materials exhibit low reported immunogenicity in autologous applications; most adverse events are limited to transient bruising or edema [73,83]. Standardization remains the principal barrier: differences in extraction, centrifugation, and dosing yield variable outcomes. Regulatory frameworks (FDA 361 vs. 351) increasingly emphasize reproducibility and validated cell characterization [94].

Proposed biological synergy model

The biological synergy model is an author-proposed, hypothesis-generating framework based on the potentially complementary mechanisms of platelet concentrates, fat-derived matrices, stem-cell-associated products, and extracellular vesicles. Direct clinical evidence showing that combining these modalities improves efficacy, safety, durability, or the need for surgery compared with established treatments is currently insufficient. Therefore, the model should be viewed as a framework for future comparative research rather than a clinical treatment algorithm. Any clinical application must follow product-specific regulatory requirements, established safety standards, and the evidence available for the individual intervention [103-106].

Global regulatory landscape

There is no single, globally applicable framework for aesthetic medicine, and the regulatory environment is diverse and often fragmented. Some nations rely on more general healthcare laws or voluntary professional recommendations for aesthetic procedures, while others have specialized rules, especially for cosmetic surgery. The regulation of minimally invasive procedures, such as Botox and fillers, which are occasionally performed by people with little to no professional medical training, is a major topic of concern [107]. Within its larger framework for human cells, tissues, and cellular and tissue-based products (HCT/Ps), the FDA regulates MSCs in the fields of aesthetics and cosmetology. The majority of MSC applications are not FDA-approved for cosmetic purposes, whereas some are approved for the treatment of specialized disorders. The FDA promotes a flexible, risk-based strategy that prioritizes safety and effectiveness, especially for products used for homologous use and with little manipulation [108,109]. Under Section 361 of the Public Health Service Act, which has less strict regulations than Section 351, products that are utilized for homologous use, that is, serve the same fundamental function in the recipient as in the donor, and that are minimally modified may be subject to control. Section 351 applies to products used for nonhomologous purposes or those that are more than minimally modified. These products require premarket approval and Investigational New Drug (IND) applications, which involve demonstrating their safety and effectiveness [108].

Across global markets, regulatory maturity varies significantly. The United States and the European Union maintain the most clearly defined frameworks, distinguishing between minimally manipulated, homologous-use biologics (361) and more-than-minimally manipulated products that require full biologic licensure (351). Countries such as the UAE, KSA, and Brazil are developing structured guidance for regenerative aesthetic materials. At the same time, India currently operates with emerging standards focused primarily on safety, sterility, and ethical sourcing. As biologics gain clinical traction, international convergence on cell characterization, donor screening, and standardized manufacturing is expected to become essential for achieving safe, reproducible outcomes.

**Figure 5:**
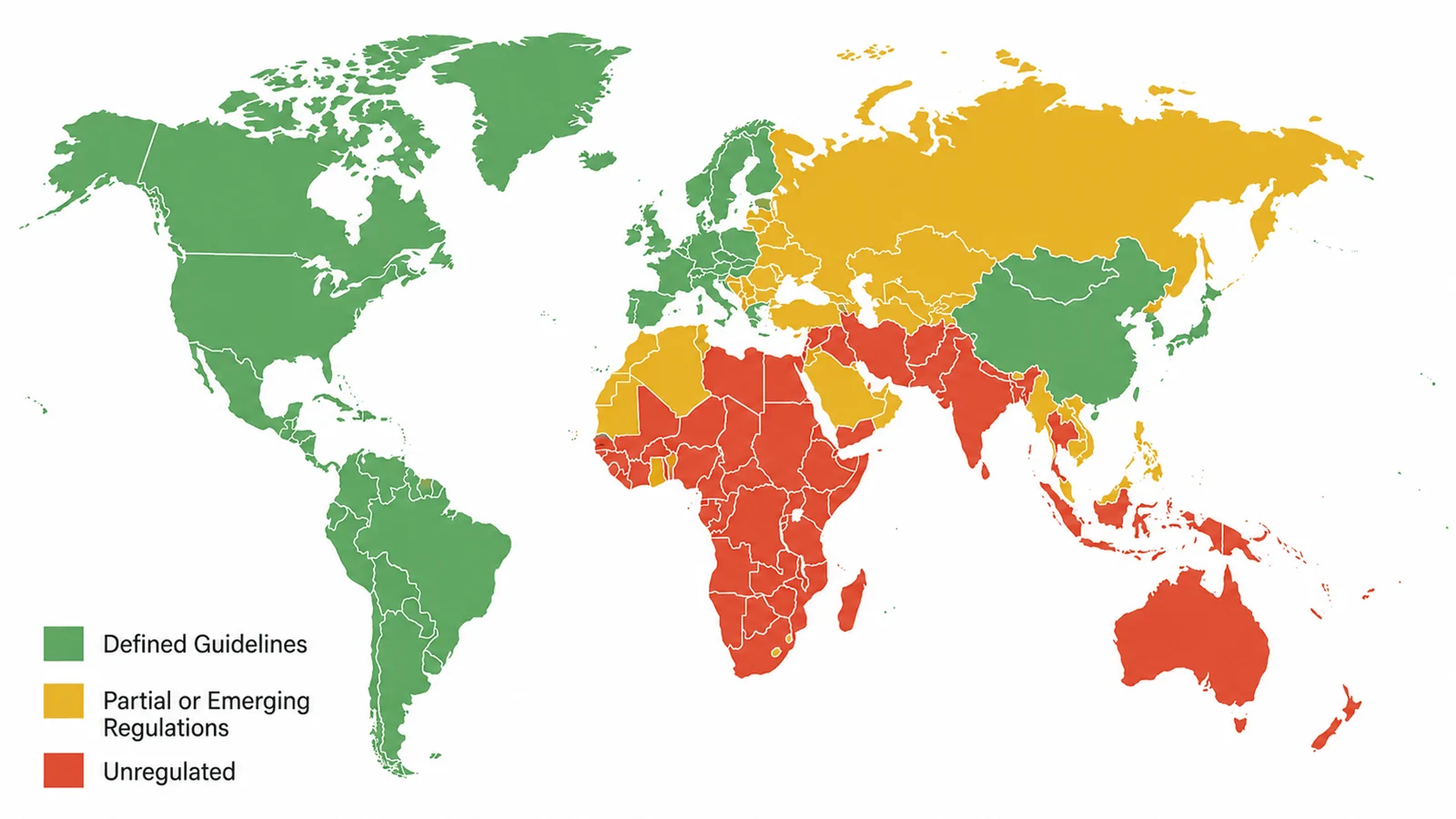
Global Regulatory Landscape of Biological Aesthetic Agents This map presents a comparative overview of regulatory maturity across countries governing the use of biological aesthetic products. Green regions indicate well-defined frameworks (e.g., USA, EU, Japan, South Korea), and yellow highlights emerging or partially developed guidelines (e.g., India, UAE, Brazil). At the same time, red areas denote unregulated or undefined sectors. Data synthesized from reports by the U.S. FDA, EMA, CDSCO, PMDA Japan, and WHO position papers on regenerative medicine [107–109]. Regulatory classifications are jurisdiction-specific and may change over time. This figure was created using BioRender (trial version, Science Suite Inc. d/b.a. BioRender, Toronto, Canada) for graphical elements. USA: United States of America; EU: European Union; UAE: United Arab Emirates; FDA: Food and Drug Administration; EMA: European Medicines Agency; CDSCO: Central Drugs Standard Control Organization; PMDA: Pharmaceuticals and Medical Devices Agency; WHO: World Health Organization.

Commercial translation and regulatory caution

Commercial platforms involving MSCs, extracellular vesicle or exosome-associated preparations, automated PRF systems, and adipose-derived matrices are available or under development. However, their composition, intended use, regulatory classification, and supporting evidence differ substantially between products and jurisdictions. Ryoncil (remestemcel-L-rknd) (Mesoblast, Inc., New York), for example, is approved by the US Food and Drug Administration for steroid-refractory acute graft-versus-host disease in pediatric patients and not for aesthetic use. This approval cannot be extrapolated to cosmetic MSC therapy. Regulatory authorities have also warned about safety concerns and unsupported claims involving unapproved human cell-, tissue-, and exosome-associated products. Commercial names are mentioned only as translational examples and not as endorsements. Manufacturer-reported claims should not be considered independent evidence of clinical efficacy.

Ryoncil®

Human MSCs, which have the capacity to inhibit the immune system, were isolated from the bone marrow of healthy adult donors and grown ex vivo. Because remestemcel-L cells do not express the major histocompatibility complex II (MHC II) molecule, they are hypoimmunogenic and do not trigger a strong host immune response when infused intravenously. Allogeneic MSC infusion may lead to (a) increased production of anti-inflammatory cytokines, including hepatocyte growth factor, prostaglandin E, and interleukin-10; (b) decreased expression of indoleamine 2,3-dioxygenase in mononuclear phagocytes, which catabolizes L-tryptophan into its pro-inflammatory metabolites; and (c) altered dendritic cell (DC) maturation and disturbance of the functions of CD8+ and CD4+ T cells as well as natural killer (NK) cells. Furthermore, after administration, pluripotent MSCs may be drawn to injured tissue and, when stimulated, differentiate into specific lineages [110,111].

ExoJuv™

ExoJuv™ (Oreon Life Science, South Korea)is a cutting-edge exosome-based regenerative treatment for skin and hair that utilizes extracellular vesicles, or exosomes, to stimulate tissue regeneration and enhance cell signaling. In addition to providing anti-inflammatory, antioxidant, and skin-brightening properties, it is formulated to address various skin issues, including fine lines, impaired skin texture, and hair loss. Healthcare or cosmetic specialists usually use direct injection or microneedling to administer ExoJuv [54,112]. Exosomes in ExoJuv are purported to promote collagen production, accelerate tissue healing, and enhance the overall vitality of the skin, resulting in a more youthful appearance [54,112].

Renewosome™

Platelet-derived exosomes are used in Renewosome™, a unique technology created by (plated)™ Skin Science (RION Aesthetics, Rochester, Minnesota) to support skin and hair renewal. These human platelet-derived exosomes are believed to promote cellular renewal, alleviate symptoms of aging, and improve the texture and tone of the skin. A major feature of (plated)™ skincare products, such as face and scalp serums, is the technology [113]. Exosomes, which are microscopic vesicles secreted by cells that contain signaling molecules and other beneficial materials, form the basis of Renewosome™ technology. Human platelets, renowned for their role in tissue regeneration and repair, are the unique source of the exosomes used in Renewosome™. By increasing collagen synthesis, reducing redness and wrinkles, enhancing tone and brightness, minimizing pore size, and protecting against environmental damage, Renewosome™ aims to revitalize the skin. Additionally, a hair serum that promotes hair growth and enhances scalp health uses the technology. A variety of (plated)™ products, including serums for hair development, post-procedure healing, and antiaging, contain Renewosome™ technology. Clinical studies have demonstrated that (plated)™ products utilizing Renewosome™ technology can improve overall skin appearance and reduce the visible signs of aging [114,115].

Vivostat PRF

A therapeutic agent called Vivostat PRF utilizes the patient's own platelets and fibrin to create a concentrated matrix that promotes wound healing and tissue regeneration. This autologous PRF is prepared by an automated instrument called the Vivostat system. It promotes healing and reduces complications in various surgical and wound care applications. Because Vivostat PRF is made from the patient's own blood, there is a lower risk of infection or rejection. High levels of platelets, which release growth factors, and a fibrin matrix, which serves as a scaffold for tissue regeneration and cell migration, are found in the PRF. For PRF preparation, the Vivostat system offers a fully automated procedure that ensures efficiency and consistency.

Numerous surgical techniques across orthopedics, neurology, and wound care utilize Vivostat PRF [116].

Advanced PRF (A-PRF)

A second-generation platelet concentrate, known as A-PRF, is utilized in dentistry and other fields to accelerate tissue regeneration. It is made from a patient's own blood and concentrates platelets and growth factors within a fibrin matrix using a modified centrifugation technique, thereby accelerating healing and reducing inflammation [117]. Because A-PRF is made from the patient's own blood, there is a lower risk of immunological or allergic reactions. Because A-PRF is made without the use of anticoagulants, it produces a denser and more stable fibrin matrix than specific previous platelet concentrates. Numerous growth factors, including PDGF, VEGF, and TGF-beta, present in A-PRF, are essential for tissue regeneration and repair. Compared to previous PRF products, A-PRF has a higher concentration of white blood cells (leukocytes). Both wound healing and immunological function depend on these leukocytes. During the healing process, the fibrin matrix in A-PRF provides structural support by serving as a scaffold for tissue growth and cell migration [117]. A-PRF+ is an improved form of A-PRF that has a denser fibrin matrix and even greater growth factor concentrations. Even greater outcomes in tissue healing and regeneration could result from this. To prolong the release of growth factors and promote tissue regeneration, A-PRF+ is designed to be more stable and remain at the wound site for an extended period [118].

Injectable PRF (i-PRF)

With a high concentration of platelets and growth factors extracted from the patient's own blood, i-PRF, a liquid form of PRF, reduces the possibility of immunological or allergic reactions. It is utilized in regenerative medicine to support tissue regeneration and healing in various fields, including dermatology, orthopedics, and dentistry [119-121]. In contrast to conventional PRF, i-PRF stays liquid for a while after preparation, making injection simpler and its use more widespread. Additionally, it contains several growth factors essential for tissue regeneration and repair, including vascular endothelial growth factor (VEGF), transforming growth factor beta (TGF-β), and platelet-derived growth factor (PDGF). Thus, the liquid i-PRF progressively solidifies into a fibrin clot, which serves as a scaffold for tissue regeneration and cell migration. Compared to other platelet concentrates, i-PRF has a higher leukocyte (white blood cell) concentration, which may enhance its antibacterial and regenerative properties. Since i-PRF preparation requires very little equipment and supplies, it is a reasonably priced choice [121,122].

GEM 21S

A growth factor-enhanced matrix called GEM 21S is utilized in dentistry to promote the regeneration of soft tissues and bone, especially in the treatment of periodontal abnormalities. It combines a growth factor, recombinant human platelet-derived growth factor (rhPDGF-BB), with a material resembling bone, beta-tricalcium phosphate (β-TCP). By promoting bone growth and tissue regeneration, this combination facilitates quicker, more efficient recovery. The two primary components of GEM 21S are (1) β-TCP, a synthetic bone graft material that serves as a scaffold for the formation of new bone and aids in blood clot stabilization, and (2) rhPDGF-BB, a purified, recombinant form of platelet-derived growth factor, a naturally occurring protein that is essential for tissue regeneration and wound healing. New bone cells can grow on the physical structure provided by β-TCP. To promote the regeneration of bone, connective tissue, and the periodontal ligament, rhPDGF-BB attracts and activates various cells, including osteoblasts (cells that produce bone), fibroblasts (cells that form connective tissue), and cementoblasts (cells that form cementum). To supply nutrition and oxygen to the healing region, rhPDGF-BB also promotes angiogenesis, the growth of new blood vessels. GEM 21S is essentially an advanced tissue-engineering technique that boosts the body's innate capacity to repair periodontal abnormalities by combining a potent growth factor with a bone graft material [122].

Renuva

Renuva is an injectable treatment that encourages the body's natural fat regeneration to restore volume in certain facial and body areas. It uses a processed human tissue matrix to provide a foundation for the development of new fat cells, offering an alternative to conventional fat grafting and artificial fillers. Renuva produces long-lasting, natural-looking outcomes by encouraging the body's own fat cells to proliferate in the treated area. Like other fillers, it is injected with a needle or cannula and has a short recovery period. Renuva can be applied to the face, hands, and body to treat cellulite dimples, skin abnormalities, and volume loss. Compared to surgical fat grafting, Renuva provides a less invasive alternative that does not require liposuction. Made from processed human tissue, Renuva contains proteins, collagen, and growth factors found in natural fat. Renuva serves as a scaffold, giving the injected area the building blocks it needs to produce new fat cells. The treated area progressively regains volume and improves in appearance as the Renuva matrix is gradually replaced by the body's own fat cells. Renuva utilizes the body's own regeneration mechanisms to offer a less invasive and natural approach to volume restoration [123].

Future research directions

AI-assisted molecular design, biomarker-guided treatment selection, tissue-engineered skin, and virtual aesthetic modeling are active areas of investigation. Their future roles in aesthetic medicine remain uncertain and will depend on analytical validation, prospective clinical trials, reproducible manufacturing, regulatory review, cost-effectiveness, and long-term safety [124-126]. The “Biological Beauty 2030” model presented in this review is an author-developed, hypothesis-generating framework rather than a prediction of future clinical practice.

**Figure 6:**
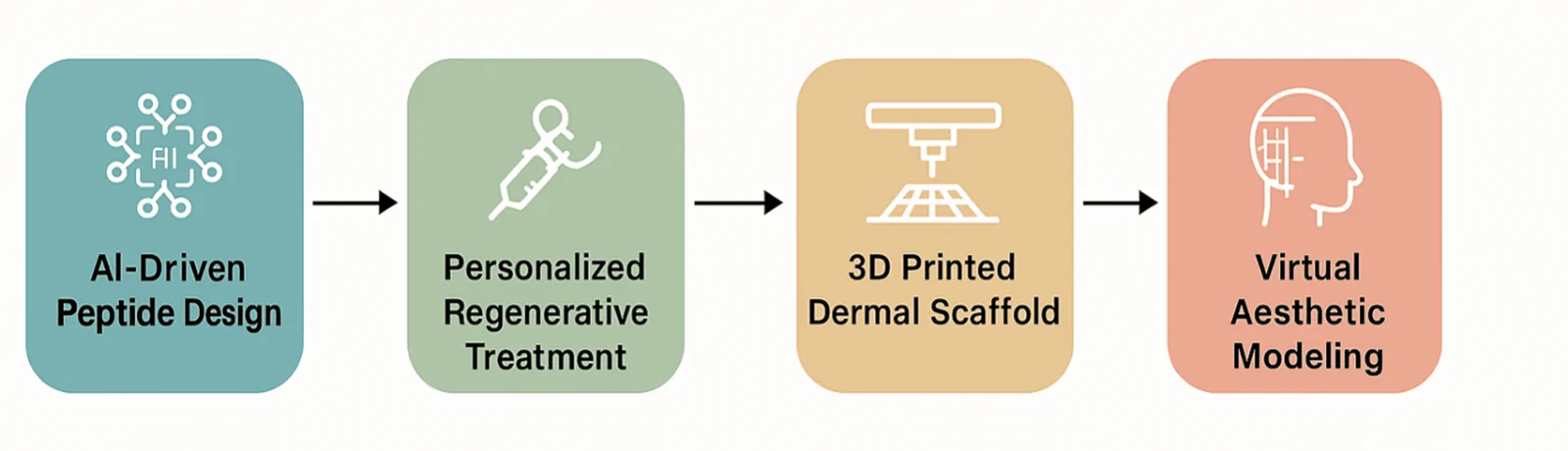
Author-Proposed Future Research Framework A conceptual framework illustrating the integration of artificial intelligence, regenerative biology, and virtual modeling in next-generation aesthetic medicine. This schematic outlines the projected paradigm of “Biological Beauty 2030,” emphasizing a data-driven, regenerative, and sustainable approach to aesthetic care. The model integrates four key pillars: (1) AI-driven peptide design for customized molecular therapy; (2) genetically and epigenetically personalized regenerative treatment; (3) 3D-printed dermal scaffolds for tissue reconstruction; and (4) virtual aesthetic modeling for predictive visualization. Adapted from emerging research trends in regenerative medicine and AI-driven bioengineering [124–126]. This hypothesis-generating model is not a forecast of clinical practice or a treatment recommendation. This figure was created using BioRender (trial version, Science Suite Inc. d/b.a. BioRender, Toronto, Canada) for graphical elements and Microsoft PowerPoint (Microsoft Corporation, Redmond, Washington) for arrangement, labeling, and formatting. Images courtesy of Dr. Mohamed Ismail.

Limitations

This narrative review has several limitations. The literature search and study selection were not systematic, and selection and publication bias cannot be excluded. The cited evidence includes in vitro and animal studies, small clinical studies, observational reports, registered trials without published results, narrative reviews, regulatory documents, and commercial sources. Mechanistic plausibility should not be interpreted as proof of clinical efficacy. Few comparative studies have evaluated multilayer combinations against established aesthetic treatments, and long-term safety, preparation methods, dosing, patient selection, and regulatory requirements remain inconsistent. Therefore, the proposed layered protocol and future framework should be interpreted as hypothesis-generating concepts rather than clinical practice guidelines.

## Conclusions

Regenerative approaches such as autologous platelet concentrates and fat-derived materials have plausible biological mechanisms and some clinical support for selected applications. Evidence for MSC-based products, exosomes, bioactive peptides, and multilayer combination protocols remains heterogeneous and is often preclinical or derived from small clinical studies. Differences in product composition, preparation, dosing, regulation, and outcome assessment prevent firm treatment recommendations. The layered model proposed in this narrative review may help organize future research but should not be interpreted as a validated clinical algorithm. Well-designed comparative trials, standardized reporting, independent safety surveillance, and compliance with jurisdiction-specific regulations are required before these approaches can be adopted more broadly.

## References

[1] Trovato F, Ceccarelli S, Michelini S (2024). Advancements in regenerative medicine for aesthetic dermatology: a comprehensive review and future trends. Cosmetics.

[2] Reddy MS, Ponnamma D, Choudhary R, Sadasivuni KK (2021). A comparative review of natural and synthetic biopolymer composite scaffolds. Polymers (Basel).

[3] Gomes C, Silva AC, Marques AC (2020). Biotechnology applied to cosmetics and aesthetic medicines. Cosmetics.

[4] Tran DK, Phuong TN, Bui NL (2023). Exploring the potential of stem cell-based therapy for aesthetic and plastic surgery. IEEE Rev Biomed Eng.

[5] Porcello A, Chemali M, Marques C (2024). Dual functionalization of hyaluronan dermal fillers with vitamin B3: efficient combination of bio-stimulation properties with hydrogel system resilience enhancement. Gels.

[6] Coffman JA, Rieger S, Rogers AN, Updike DL, Yin VP (2016). Comparative biology of tissue repair, regeneration and aging. NPJ Regen Med.

[7] Goldie K (2023). The evolving field of regenerative aesthetics. J Cosmet Dermatol.

[8] Shah K, Verma K, Trivedi J, Mehta S, Kumar S, Dhameliya T (2024). Natural products for cosmeceuticals. Bioprospecting of Natural Sources for Cosmeceuticals.

[9] Czop JK, Jałowska M (2023). Stem cells in plastic surgery and aesthetic medicine. Postepy Dermatol Alergol.

[10] Zhang H, Xiao X, Wang L, Shi X, Fu N, Wang S, Zhao RC (2024). Human adipose and umbilical cord mesenchymal stem cell-derived extracellular vesicles mitigate photoaging via TIMP1/Notch1. Signal Transduct Target Ther.

[11] de Sanctis Pecora C, Corduff N, Carroll J, Muniz M, Sattler S (2025). Multimodal treatment combinations and layering to restructure the aging face: recommendations from an expert panel. Plast Reconstr Surg Glob Open.

[12] Cotofana S, Fratila AA, Schenck TL, Redka-Swoboda W, Zilinsky I, Pavicic T (2016). The anatomy of the aging face: a review. Facial Plast Surg.

[13] Hernandez C, Viscomi B, Faria G, Vasconcelos R, Schneider C, Moreno J, Muniz M (2024). Multilayer technique using calcium hydroxylapatite biostimulation with different dilutions in the lateral face. Aesthet Surg J Open Forum.

[14] de Sanctis Pecora C (2024). The anatomical layering assessment: the construction of beauty. Clin Cosmet Investig Dermatol.

[15] Cotofana S, Lachman N (2019). Anatomy of the facial fat compartments and their relevance in aesthetic surgery. J Dtsch Dermatol Ges.

[16] Bagheri SC, Bohluli B, Consky EK (2018). Current techniques in fat grafting. Atlas Oral Maxillofac Surg Clin North Am.

[17] Coleman SR, Katzel EB (2015). Fat grafting for facial filling and regeneration. Clin Plast Surg.

[18] Rohrich RJ, Pessa JE (2007). The fat compartments of the face: anatomy and clinical implications for cosmetic surgery. Plast Reconstr Surg.

[19] Narasimhan K, Ramanadham S, Rohrich RJ (2015). Face lifting in the massive weight loss patient: modifications of our technique for this population. Plast Reconstr Surg.

[20] Haykal D, Flament F, Mora P, Balooch G, Cartier H (2025). Unlocking longevity in aesthetic dermatology: epigenetics, aging, and personalized care. Int J Dermatol.

[21] Kapoor KM, Saputra DI, Porter CE (2021). Treating aging changes of facial anatomical layers with hyaluronic acid fillers. Clin Cosmet Investig Dermatol.

[22] Landau M, Anand CV, Besins T (2017). First consensus on primary prevention and early intervention in aesthetic medicine. J Drugs Dermatol JDD.

[23] Suwanchinda A, Webb KL, Rudolph C (2018). The posterior temporal supraSMAS minimally invasive lifting technique using soft-tissue fillers. J Cosmet Dermatol.

[24] Pountos I, Panteli M, Lampropoulos A, Jones E, Calori GM, Giannoudis PV (2016). The role of peptides in bone healing and regeneration: a systematic review. BMC Med.

[25] Eun SC (2014). Stem cell and research in plastic surgery. J Korean Med Sci.

[26] Jin Y, Li S, Yu Q, Chen T, Liu D (2023). Application of stem cells in regeneration medicine. MedComm (2020).

[27] Frame JD, Connolly C (2015). Framing the breast. Aesthet Surg J.

[28] Conese M, Annacontini L, Carbone A (2020). The role of adipose-derived stem cells, dermal regenerative templates, and platelet-rich plasma in tissue engineering-based treatments of chronic skin wounds. Stem Cells Int.

[29] Zheng Y, Wei Z, Wang T (2023). MOTS-c: a promising mitochondrial-derived peptide for therapeutic exploitation. Front Endocrinol (Lausanne).

[30] White C, Brahs A, Dorton D, Witfill K (2021). Platelet-rich plasma: a comprehensive review of emerging applications in medical and aesthetic dermatology. J Clin Aesthet Dermatol.

[31] Lin MY, Lin CS, Hu S, Chung WH (2020). Progress in the use of platelet-rich plasma in aesthetic and medical dermatology. J Clin Aesthet Dermatol.

[32] Grecu AF, Reclaru L, Ardelean LC, Nica O, Ciucă EM, Ciurea ME (2019). Platelet-rich fibrin and its emerging therapeutic benefits for musculoskeletal injury treatment. Medicina (Kaunas).

[33] Irma J, Kartasasmita AS, Kartiwa A, Irfani I, Rizki SA, Onasis S (2025). From growth factors to structure: PDGF and TGF-β in granulation tissue formation. A literature review. J Cell Mol Med.

[34] Xiong M, Zhang Q, Hu W (2021). The novel mechanisms and applications of exosomes in dermatology and cutaneous medical aesthetics. Pharmacol Res.

[35] Rambhia PH, Turner L, Ugonabo N, Chapas A (2022). Muscle stimulation for aesthetic body shaping: a comprehensive and critical review. Dermatol Surg.

[36] Rudnicki PA, Purt B, True D, Siordia H, Lohmeier S, Chan RK (2020). Single-stage composite skin reconstruction using a dermal regeneration template. Plast Reconstr Surg Glob Open.

[37] Villarreal-Gómez LJ, Origel-Lucio S, Hernández-Hernández DA (2025). Use of exosomes for cosmetics applications. Cosmetics.

[38] Santos LC, Lana GL, Santos GS (2024). The biological role of platelet derivatives in regenerative aesthetics. Int J Mol Sci.

[39] Juhl P, Bondesen S, Hawkins CL, Karsdal MA, Bay-Jensen AC, Davies MJ, Siebuhr AS (2020). Dermal fibroblasts have different extracellular matrix profiles induced by TGF-β, PDGF and IL-6 in a model for skin fibrosis. Sci Rep.

[40] Karimi K, Rockwell H (2019). The benefits of platelet-rich fibrin. Facial Plast Surg Clin North Am.

[41] Pickart L, Vasquez-Soltero J, Margolina A (2015). GHK-Cu may prevent oxidative stress in skin by regulating copper and modifying expression of numerous antioxidant genes. Cosmetics.

[42] Avci P, Gupta A, Sadasivam M, Vecchio D, Pam Z, Pam N, Hamblin MR (2013). Low-level laser (light) therapy (LLLT) in skin: stimulating, healing, restoring. Semin Cutan Med Surg.

[43] Arora G, Arora S (2023). Medical aesthetics - current trends and a review of its applications. Indian Dermatol Online J.

[44] Gavish L, Houreld NN (2019). Therapeutic efficacy of home-use photobiomodulation devices: a systematic literature review. Photobiomodul Photomed Laser Surg.

[45] Thakur A, Shah D, Rai D (2023). Therapeutic values of exosomes in cosmetics, skin care, tissue regeneration, and dermatological diseases. Cosmetics.

[46] Masaki H (2010). Role of antioxidants in the skin: anti-aging effects. J Dermatol Sci.

[47] Kohen R (1999). Skin antioxidants: their role in aging and in oxidative stress - new approaches for their evaluation. Biomed Pharmacother.

[48] Hussen NHA, Abdulla SK, Ali NM (2025). Role of antioxidants in skin aging and the molecular mechanism of ROS: a comprehensive review. Asp Mol Med.

[49] Hakozaki T, Minwalla L, Zhuang J (2002). The effect of niacinamide on reducing cutaneous pigmentation and suppression of melanosome transfer. Br J Dermatol.

[50] Piao MJ, Kang KA, Zhen AX (2019). Particulate matter 2.5 mediates cutaneous cellular injury by inducing mitochondria-associated endoplasmic reticulum stress: protective effects of ginsenoside Rb1. Antioxidants (Basel).

[51] Mokalled MH, Poss KD (2018). A regeneration toolkit. Dev Cell.

[52] Mason C, Manzotti E (2010). Regenerative medicine cell therapies: numbers of units manufactured and patients treated between 1988 and 2010. Regen Med.

[53] Dai R, Wang Z, Samanipour R, Koo KI, Kim K (2016). Adipose-derived stem cells for tissue engineering and regenerative medicine applications. Stem Cells Int.

[54] Zhang B, Gong J, He L, Khan A, Xiong T, Shen H, Li Z (2022). Exosomes based advancements for application in medical aesthetics. Front Bioeng Biotechnol.

[55] Olumesi KR, Goldberg DJ (2023). A review of exosomes and their application in cutaneous medical aesthetics. J Cosmet Dermatol.

[56] Pintea A, Manea A, Pintea C (2025). Peptides: emerging candidates for the prevention and treatment of skin senescence: a review. Biomolecules.

[57] Mohtashami Z, Singh MK, Salimiaghdam N, Ozgul M, Kenney MC (2022). MOTS-c, the most recent mitochondrial derived peptide in human aging and age-related diseases. Int J Mol Sci.

[58] Brunner LM, He Y, Cousin N (2023). Promotion of lymphangiogenesis by targeted delivery of VEGF-C improves diabetic wound healing. Cells.

[59] Papadopoulos KS, Piperi C, Korkolopoulou P (2024). Clinical applications of adipose-derived stem cell (ADSC) exosomes in tissue regeneration. Int J Mol Sci.

[60] Salibian AA, Widgerow AD, Abrouk M, Evans GR (2013). Stem cells in plastic surgery: a review of current clinical and translational applications. Arch Plast Surg.

[61] Bharti D, Shivakumar SB, Park JK (2018). Comparative analysis of human Wharton's jelly mesenchymal stem cells derived from different parts of the same umbilical cord. Cell Tissue Res.

[62] Mehrabani D, Mehrabani G, Zare S, Manafi A (2013). Adipose-derived stem cells (ADSC) and aesthetic surgery: a mini review. World J Plast Surg.

[63] Gaur M, Dobke M, Lunyak VV (2017). Mesenchymal stem cells from adipose tissue in clinical applications for dermatological indications and skin aging. Int J Mol Sci.

[64] Nagamura-Inoue T, He H (2014). Umbilical cord-derived mesenchymal stem cells: their advantages and potential clinical utility. World J Stem Cells.

[65] Xie Q, Liu R, Jiang J (2020). What is the impact of human umbilical cord mesenchymal stem cell transplantation on clinical treatment?. Stem Cell Res Ther.

[66] Zhao C, Zhang L, Kong W (2015). Umbilical cord-derived mesenchymal stem cells inhibit cadherin-11 expression by fibroblast-like synoviocytes in rheumatoid arthritis. J Immunol Res.

[67] Sun X, Hao H, Han Q (2017). Human umbilical cord-derived mesenchymal stem cells ameliorate insulin resistance by suppressing NLRP3 inflammasome-mediated inflammation in type 2 diabetes rats. Stem Cell Res Ther.

[68] Gubert F, da Silva JS, Vasques JF (2021). Mesenchymal stem cells therapies on fibrotic heart diseases. Int J Mol Sci.

[69] Ratti M, Lampis A, Ghidini M, Salati M, Mirchev MB, Valeri N, Hahne JC (2020). microRNAs (miRNAs) and long non-coding RNAs (lncRNAs) as new tools for cancer therapy: first steps from bench to bedside. Target Oncol.

[70] Hsieh JY, Fu YS, Chang SJ, Tsuang YH, Wang HW (2010). Functional module analysis reveals differential osteogenic and stemness potentials in human mesenchymal stem cells from bone marrow and Wharton's jelly of umbilical cord. Stem Cells Dev.

[71] Mazini L, Rochette L, Amine M, Malka G (2019). Regenerative capacity of adipose derived stem cells (ADSCs), comparison with mesenchymal stem cells (MSCs). Int J Mol Sci.

[72] Kagiya G, Ogawa R, Matsumoto T (2025). Real-time imaging reveals radiation-induced intratumor apoptosis via nutrient and oxygen deprivation following vascular damage. Mol Ther Oncol.

[73] Zhang Y, Liu Y, Liu H, Tang WH (2019). Exosomes: biogenesis, biologic function and clinical potential. Cell Biosci.

[74] Chen C, Zhang Z, Gu X, Sheng X, Xiao L, Wang X (2023). Exosomes: New regulators of reproductive development. Mater Today Bio.

[75] Lau NC, Yam JW (2023). From exosome biogenesis to absorption: key takeaways for cancer research. Cancers (Basel).

[76] Hajialiasgary Najafabadi A, Soheilifar MH, Masoudi-Khoram N (2024). Exosomes in skin photoaging: biological functions and therapeutic opportunity. Cell Commun Signal.

[77] Lee YS (2025). Regenerative skin remodeling through exosome-based therapy: a case study demonstrating 21-month sustained outcomes in pore size, erythema, and hyperpigmentation. Dermatol Ther (Heidelb).

[78] Saluja H, Dehane V, Mahindra U (2011). Platelet-Rich fibrin: A second generation platelet concentrate and a new friend of oral and maxillofacial surgeons. Ann Maxillofac Surg.

[79] Narayanaswamy R, Patro BP, Jeyaraman N (2023). Evolution and clinical advances of platelet-rich fibrin in musculoskeletal regeneration. Bioengineering (Basel).

[80] Lambrichts I, Wolfs E, Bronckaers A, Gervois P, Vangansewinkel T (2023). The effect of leukocyte- and platelet-rich fibrin on central and peripheral nervous system neurons-implications for biomaterial applicability. Int J Mol Sci.

[81] Jia K, You J, Zhu Y (2024). Platelet-rich fibrin as an autologous biomaterial for bone regeneration: mechanisms, applications, optimization. Front Bioeng Biotechnol.

[82] Dashore S, Chouhan K, Nanda S, Sharma A (2021). Platelet-rich fibrin, preparation and use in dermatology. Indian Dermatol Online J.

[83] Kim JY, Kim NH (2023). New therapeutic approaches to the treatment of dyslipidemia 1: ApoC-III and ANGPTL3. J Lipid Atheroscler.

[84] Pavlovic V, Ciric M, Jovanovic V, Trandafilovic M, Stojanovic P (2021). Platelet-rich fibrin: basics of biological actions and protocol modifications. Open Med (Wars).

[85] Fan Y, Perez K, Dym H (2020). Clinical uses of platelet-rich fibrin in oral and maxillofacial surgery. Dent Clin North Am.

[86] Kachooeian M, Mousivand Z, Sharifikolouei E, Shirangi M, Firoozpour L, Raoufi M, Sharifzadeh M (2022). Matrixyl patch vs Matrixyl cream: a comparative in vivo investigation of Matrixyl (MTI) effect on wound healing. ACS Omega.

[87] Olsson SE, Sreepad B, Lee T, Fasih M, Fijany A (2024). Public interest in acetyl hexapeptide-8: longitudinal analysis. JMIR Dermatol.

[88] Gorouhi F, Maibach HI (2009). Role of topical peptides in preventing or treating aged skin. Int J Cosmet Sci.

[89] Gold MH, Fagien S, Rohrich RJ (2024). A multicenter pilot study of a novel allograft adipose matrix in Malar and Prejowl volume restoration. Plast Reconstr Surg Glob Open.

[90] Bi X, Li Y, Dong Z (2021). Recent developments in extracellular matrix remodeling for fat grafting. Front Cell Dev Biol.

[91] Ding P, Lu E, Li G, Sun Y, Yang W, Zhao Z (2022). Research progress on preparation, mechanism, and clinical application of nanofat. J Burn Care Res.

[92] La Padula S, Ponzo M, Lombardi M (2023). Nanofat in plastic reconstructive, regenerative, and aesthetic surgery: a review of advancements in face-focused applications. J Clin Med.

[93] Schultz KP, Raghuram A, Davis MJ, Abu-Ghname A, Chamata E, Rohrich RJ (2020). Fat grafting for facial rejuvenation. Semin Plast Surg.

[94] Jo H, Brito S, Kwak BM, Park S, Lee MG, Bin BH (2021). Applications of mesenchymal stem cells in skin regeneration and rejuvenation. Int J Mol Sci.

[95] Banihashemi M, Zabolinejad N, Salehi M, Hamidi Alamdari D, Nakhaizadeh S (2021). Platelet-rich plasma use for facial rejuvenation: a clinical trial and review of current literature. Acta Biomed.

[96] Mahmoodabadi RA, Golafshan HA, Pezeshkian F, Shahriarirad R, Namazi MR (2023). Evaluation of the effect of platelet-rich fibrin matrix in the correction of periorbital wrinkles: an experimental clinical trial. Dermatol Pract Concept.

[97] Domaszewska-Szostek A, Krzyżanowska M, Polak A, Puzianowska-Kuźnicka M (2025). Effectiveness of extracellular vesicle application in skin aging treatment and regeneration: do we have enough evidence from clinical trials?. Int J Mol Sci.

[98] Geusens B, Haykal D (2025). Genetic profiling and precision skin care: a review. Front Genet.

[99] Rani A, Kaur G, Kumar R (2025). Role of DNA methylation in the pathogenesis of skin disorders: mechanisms, inhibitors of methylation-related enzyme, and molecular docking studies. Biomed Res Int.

[100] Riancho J, Del Real A, Riancho JA (2016). How to interpret epigenetic association studies: a guide for clinicians. Bonekey Rep.

[101] Rivera CM, Ren B (2013). Mapping human epigenomes. Cell.

[102] Schu S, Nosov M, O'Flynn L (2012). Immunogenicity of allogeneic mesenchymal stem cells. J Cell Mol Med.

[103] Moll G, Hoogduijn MJ, Ankrum JA (2020). Editorial: safety, efficacy and mechanisms of action of mesenchymal stem cell therapies. Front Immunol.

[104] Kale P, Patel H, Jaiswal AM (2024). Mechanisms, efficacy, and clinical applications of platelet-rich plasma in tendinopathy: a comprehensive review. Cureus.

[105] (2017). Platelet rich fibrin: a second-generation platelet concentrate. Platelet Rich Fibrin in Regenerative Dentistry: Biological Background and Clinical Indications.

[106] Bielecki T, Dohan Ehrenfest DM (2012). Platelet-rich plasma (PRP) and platelet-rich fibrin (PRF): surgical adjuvants, preparations for in situ regenerative medicine and tools for tissue engineering. Curr Pharm Biotechnol.

[107] Gopalan N (2024). Ethical and regulatory gaps in aesthetic medical practice in top Asian medical tourism destinations. Asian Bioeth Rev.

[108] Yano K, Speidel AT, Yamato M (2018). Four Food and Drug Administration draft guidance documents and the REGROW Act: a litmus test for future changes in human cell- and tissue-based products regulatory policy in the United States?. J Tissue Eng Regen Med.

[109] Fernández-Garza LE, Barrera-Barrera SA, Barrera-Saldaña HA (2023). Mesenchymal stem cell therapies approved by regulatory agencies around the world. Pharmaceuticals (Basel).

[110] Ullah I, Subbarao RB, Rho GJ (2015). Human mesenchymal stem cells - current trends and future prospective. Biosci Rep.

[111] Wang LT, Ting CH, Yen ML, Liu KJ, Sytwu HK, Wu KK, Yen BL (2016). Human mesenchymal stem cells (MSCs) for treatment towards immune- and inflammation-mediated diseases: review of current clinical trials. J Biomed Sci.

[112] Vyas KS, Kaufman J, Munavalli GS, Robertson K, Behfar A, Wyles SP (2023). Exosomes: the latest in regenerative aesthetics. Regen Med.

[113] Proffer SL, Paradise CR, DeGrazia E (2022). Efficacy and tolerability of topical platelet exosomes for skin rejuvenation: six-week results. Aesthet Surg J.

[114] Lee KW, Chan LK, Hung LC, Phoebe LK, Park Y, Yi KH (2024). Clinical applications of exosomes: a critical review. Int J Mol Sci.

[115] Chen Y, Qi W, Wang Z, Niu F (2025). Exosome source matters: a comprehensive review from the perspective of diverse cellular origins. Pharmaceutics.

[116] Bayer A, Höntsch G, Kaschwich M (2020). Vivostat platelet-rich fibrin® for complicated or chronic wounds—a pilot study. Biomedicines.

[117] Dohan DM, Choukroun J, Diss A, Dohan SL, Dohan AJ, Mouhyi J, Gogly B (2006). Platelet-rich fibrin (PRF): a second-generation platelet concentrate. Part I: technological concepts and evolution. Oral Surg Oral Med Oral Pathol Oral Radiol Endod.

[118] Sterczała B, Chwiłkowska A, Szwedowicz U, Kobielarz M, Chwiłkowski B, Dominiak M (2022). Impact of APRF+ in combination with autogenous fibroblasts on release growth factors, collagen, and proliferation and migration of gingival fibroblasts: an in vitro study. Materials (Basel).

[119] Egle K, Salma I, Dubnika A (2021). From blood to regenerative tissue: how autologous platelet-rich fibrin can be combined with other materials to ensure controlled drug and growth factor release. Int J Mol Sci.

[120] Liu M, Liu Y, Luo F (2023). The role and mechanism of platelet-rich fibrin in alveolar bone regeneration. Biomed Pharmacother.

[121] Costa FR, de Souza SA, Martins RA (2025). The role of injectable platelet-rich fibrin in orthopedics: where do we stand?. Curr Issues Mol Biol.

[122] El Serafy MEH, Razzak MYAE, Shoukheba MYM (2024). Growth factor-enhanced matrix combined either with free connective tissue graft or with collagen membrane in the treatment of Miller class II gingival recession. Tanta Dent J.

[123] (2025). Renuva®: our tissues & products. https://www.mtfbiologics.org/our-products/detail/renuva.

[124] Merino M, Tornero-Aguilera JF, Rubio-Zarapuz A, Villanueva-Tobaldo CV, Martín-Rodríguez A, Clemente-Suárez VJ (2024). Body perceptions and psychological well-being: a review of the impact of social media and physical measurements on self-esteem and mental health with a focus on body image satisfaction and its relationship with cultural and gender factors. Healthcare (Basel).

[125] Nissan N, Allen MC, Sabatino D, Biggar KK (2024). Future perspective: harnessing the power of artificial intelligence in the generation of new peptide drugs. Biomolecules.

[126] Dutra Alves NS, Reigado GR, Santos M (2025). Advances in regenerative medicine-based approaches for skin regeneration and rejuvenation. Front Bioeng Biotechnol.

